# NDRG1 facilitates the replication and persistence of Kaposi’s sarcoma-associated herpesvirus by interacting with the DNA polymerase clamp PCNA

**DOI:** 10.1371/journal.ppat.1007628

**Published:** 2019-02-27

**Authors:** Fang Zhang, Deguang Liang, Xiaoxi Lin, Zhe Zou, Rui Sun, Xing Wang, Xiaozhen Liang, Kenneth M. Kaye, Ke Lan

**Affiliations:** 1 CAS Key Laboratory of Molecular Virology & Immunology, Institute Pasteur of Shanghai, Chinese Academy of Sciences, University of Chinese Academy of Sciences, Shanghai, China; 2 Department of Medicine, Brigham and Women’s Hospital and Harvard Medical School, Boston, MA, United States of America; 3 State Key Laboratory of Virology, College of Life Sciences, Medical Research Institute, Wuhan University, Wuhan, China; University of North Carolina at Chapel Hill, UNITED STATES

## Abstract

Kaposi’s sarcoma-associated herpesvirus (KSHV) latently infects host cells and establishes lifelong persistence as an extra-chromosomal episome in the nucleus. To persist in proliferating cells, the viral genome typically replicates once per cell cycle and is distributed into daughter cells. This process involves host machinery utilized by KSHV, however the underlying mechanisms are not fully elucidated. In present study, we found that N-Myc downstream regulated gene 1 (NDRG1), a cellular gene known to be non-detectable in primary B cells and endothelial cells which are the major cell types for KSHV infection *in vivo*, was highly upregulated by KSHV in these cells. We further demonstrated that the high expression of NDRG1 was regulated by latency-associated nuclear antigen (LANA), the major viral latent protein which tethers the viral genome to host chromosome and plays an essential role in viral genome maintenance. Surprisingly, knockdown of NDRG1 in KSHV latently infected cells resulted in a significant decrease of viral genome copy number in these cells. Interestingly, NDRG1 can directly interact with proliferating cell nuclear antigen (PCNA), a cellular protein which functions as a DNA polymerase clamp during DNA replication. Intriguingly, we found that NDRG1 forms a complex with LANA and PCNA and serves as a scaffold protein bridging these two proteins. We further demonstrated that NDRG1 is critical for mediating LANA to recruit PCNA onto terminal repeat (TR) of KSHV genome, and facilitates viral DNA replication and episome persistence. Taken together, our findings suggest that NDRG1 plays an important role in KSHV viral genome replication, and provide new clues for understanding of KSHV persistence.

## Author summary

KSHV latently infects cells and establishes lifelong persistence, but the underlying mechanisms of this process has not been fully elucidated. Here, we find a novel host protein NDRG1 is highly up-regulated by KSHV infection and the viral protein LANA is essential in this process. NDRG1 is a multiple functional protein, but the role in KSHV infection remains unknown. Our findings show that NDRG1 functions as a scaffold protein that forms a complex with PCNA and LANA, thereby helping LANA load PCNA onto the viral genome and facilitating the replication and persistence of KSHV. Since NDRG1 is non-detectable in primary B cells and endothelial cells, which are the major cell types susceptible to KSHV infection *in vivo*, NDRG1 might be a candidate of therapeutic target for inhibition of KSHV persistence in malignant cells.

## Introduction

Kaposi’s sarcoma-associated herpesvirus (KSHV), a human oncogenic DNA gammaherpesvirus, is known for its causal association with human cancers, including endothelial-derived Kaposi’s sarcoma (KS), a B cell malignancy named primary effusion lymphoma (PEL), and a plasmablastic form of the B lymphoproliferative disorder named multicentric Castleman disease (MCD) [1–4]. KSHV infection of host cells is predominantly latent, and the virus establishes lifelong persistence of its genome in proliferating cells, which contributes to tumorigenesis. During latency, KSHV exists as a circular extrachromosomal episome tethered to the host chromosome [5–8]. To persist in cells, KSHV typically replicates once, accompanied by host replication, and is distributed into daughter cells along with the host chromosomes [5,6,8–12]. KSHV-positive PEL cells, such as BCBL1, BC3, and JSC1, are cultured cell lines established from KSHV-infected PEL samples, and there are multiple copies of viral episomes in these cells, ranging from approximately 50 to 200 per cell. The copy number of the KSHV genome remains constant with cell division, suggesting that there are mechanisms by which KSHV persists in these cells.

KSHV utilizes various viral and cellular factors for perpetuation in host cells. An indispensable viral factor is the latency-associated nuclear antigen (LANA), one of the few viral proteins expressed during latency, which is required and sufficient for KSHV episome persistence in the absence of other viral genes. LANA, encoded by the KSHV open reading frame 73 (ORF73), is an 1162-amino-acid protein. This protein tethers viral episomes to cellular chromosomes and ensures episomal DNA replication during each cell division event and segregation of viral DNA into progeny nuclei [6,8,10,11,13]. The C terminus of LANA directly binds to the KSHV latent replication origin-terminal repeat (TR) DNA to mediate viral DNA replication [5,8,14–19]. The N terminus of LANA interacts with the host chromosome and is critical for the efficiency of LANA-mediated viral DNA replication and episome persistence [20–24]. However, LANA lacks the enzymatic activity required for DNA replication. To achieve this function, LANA recruits numerous cellular replication factors, such as the origin recognition complex (ORC1-6), replication factor C (RFC), minichromosome maintenance complex (MCM), topoisomerase II beta (TopoII beta), structure-specific recognition protein 1 (SSRP1), and proliferating cell nuclear antigen (PCNA) [19,23,25–28].

PCNA is a DNA clamp that is highly conserved in eukaryotic species and is essential for DNA replication, acting as a processivity factor for DNA polymerase epsilon. PCNA encircles the DNA and executes its processivity as a scaffold, enrolling proteins that participate in DNA synthesis or repair [29–31]. Recently, it was found that PCNA is loaded onto the KSHV TR region by LANA, which is a rate-limiting step in viral DNA replication [23]. The recruitment by LANA of PCNA for loading onto the KSHV latent replication origin allows increased efficiency of viral replication and persistence. Although LANA does not interact with PCNA directly, this antigen recruits PCNA via several adaptors, such as the replication factor RFC and the cellular mitotic kinase Bub1, to assist viral replication and persistence during latency. Depletion of RFC or Bub1 has a negative impact on LANA’s ability to replicate and maintain viral episomes in KSHV-infected cells or in cells containing the TR plasmid, thus leading to loss of virus [23,27].

To investigate how KSHV utilizes cellular factors to maintain viral persistence during latency, we compared primary rat embryonic metanephric mesenchymal precursor cells (MM cells) and KSHV-immortalized MM cells (KMM cells) [32] and identified a host protein, named N-Myc downstream regulated gene 1 (NDRG1), that was distinctly upregulated in KMM cells. NDRG1 is a multifunctional protein that is involved in cell growth, differentiation, development, stress response, etc. [33–35]. However, whether NDRG1 associates with KSHV during infection and plays a role in KSHV persistence has not yet been determined. NDRG1 is widely expressed in numerous human tissues but is nondetectable in certain cell types, such as primary B cells and endothelial cells [36]. Remarkably, the prevailing view is that KS is derived from endothelial cells and that PEL is derived from B cells that are permissive to KSHV infection [37,38], suggesting that KSHV might specifically upregulate NDRG1 in these cells to facilitate infection. In this study, we demonstrated that NDRG1 is highly expressed in KSHV-positive cells, including KSHV-infected KS and PEL cells. We further demonstrated that the high expression of NDRG1 in KSHV-positive cells is induced by KSHV infection and that LANA is essential for promotion and maintenance of NDRG1 expression in KSHV-infected cells. Surprisingly, knockdown of NDRG1 dramatically reduces the viral copy numbers and leads to loss of viral genomes in cells latently infected with KSHV. Interestingly, we found that NDRG1 directly interacts with PCNA and that NDRG1 also forms a complex with LANA and PCNA. We further showed that NDRG1 acts as a scaffold protein, mediating the recruitment of PCNA by LANA onto the KSHV TR, leading to the promotion of viral DNA replication and episome persistence during latency.

## Results

### NDRG1 is highly expressed in KSHV-positive cells

**Fig 1:**
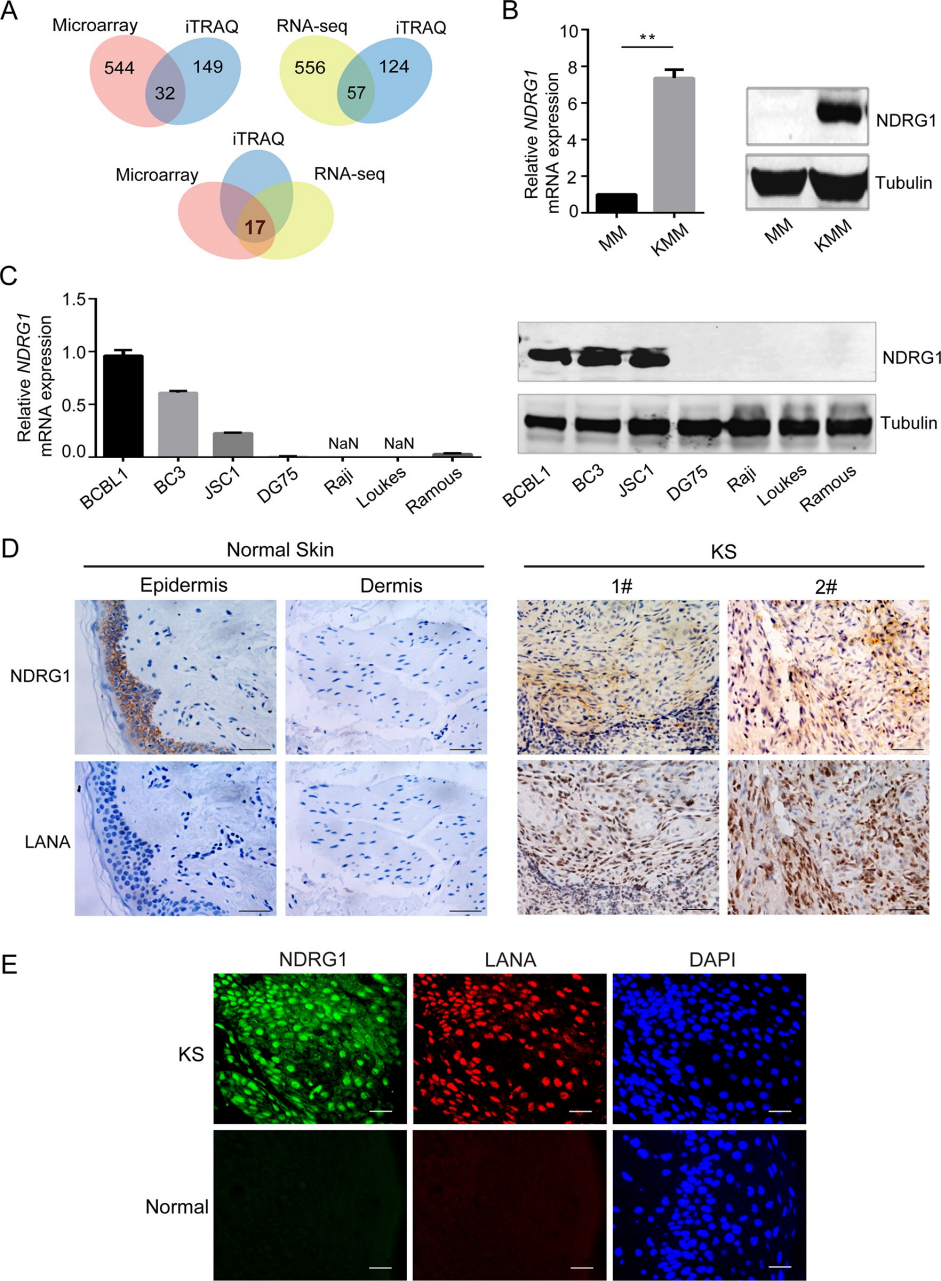
NDRG1 is highly expressed in KSHV positive cells. (A) Venn diagram showing the overlaps of differentially expressed candidate genes in KMM and MM cells. (B) NDRG1 expression in KMM and MM cells was measured by qPCR (left) and western blotting (right). qPCR data were normalized to endogenous actin expression and were shown as mean±SD, n = 3, **p<0.01. (C) NDRG1 expression in KSHV positive human B cells (BCBL1, BC3, and JSC1) and KSHV negative human B cells (DG75, Raji, Loukes, and Ramous) was detected by qPCR (left) and western blotting (right). The RNA level of NDRG1 was normalized to endogenous actin in each group, and the NDRG1 level in BCBL1 cells was set as 1.0. Data were shown as mean ± SD, n = 3. (D) Expression of NDRG1 and LANA were detected in consecutive sections of KS tissues and normal skin tissues by immunohistochemistry. Representative images of the IHC staining were shown. Scale bars represent 50 μm. (E) Immunofluorescence staining of KS tissue and normal skin tissue sections were performed. Green color indicates NDRG1, red color indicates LANA, and blue color indicates cell nuclei. Scale bars represent 25 μm.

KSHV utilizes various cellular factors to persist in proliferating host cells [10,11]. To investigate the underlying mechanisms, we performed RNA-seq, microarray analysis and iTRAQ to identify factors that may affect the persistence of viral latency using primary rat embryonic MM cells and KMM cells as a pair of uninfected and KSHV-infected cells, respectively [32]. We performed an integrative analysis of transcriptomic and proteomic changes and screened out a series of differentially expressed candidate genes. Thirty-two genes (S1 Table) were identified as being differentially expressed at both the RNA and protein levels by comparing the microarray and iTRAQ databases. Fifty-seven genes (S2 Table) were generated by assessing the RNA-seq and iTRAQ data, and seventeen genes (S3 Table) were screened out by analyzing the microarray, RNA-seq, and iTRAQ databases (Fig 1A). To verify the candidates, we tested these genes in KMM and MM cells via qPCR (S1 Fig). Surprisingly, we found that a cellular gene named NDRG1, known to be nondetectable in primary B cells and endothelial cells, which are permissive to KSHV infection [37], was significantly upregulated in KMM cells at the RNA level (Fig 1B). Consistently, NDRG1, which was detected approximately 43kDa, was markedly upregulated in KMM cells at protein level, while it was non-detectable in MM cells (Fig 1B). These results proved that NDRG1 is at higher levels in KSHV-positive KMM cells than in KSHV-negative MM cells, indicating that NDRG1 might play a role in viral persistence.

We next tested the endogenous expression level of NDRG1 in KSHV-positive human PEL cell lines (BCBL1, BC3, and JSC1), which were established from KSHV-infected PEL tissue samples [39–41], and KSHV-negative human B lymphoma cell lines (DG75, Raji, Loukes, and Ramous). The results were consistent with those observed in rat cells. NDRG1 was highly expressed in all KSHV-positive B cells but barely detected in KSHV-negative B cells at both the RNA and protein levels (Fig 1C). Given that NDRG1 is known to be nondetectable in primary B cells [36], the results suggested that the presence of NDRG1 in KSHV-positive cells might be important for persistent KSHV infection in not only rat cells but also human cells.

We further examined NDRG1 expression in KSHV-positive KS tumor samples and KSHV-negative normal skin tissues by immunohistochemical assays. LANA is one of the major latent viral proteins expressed in KS spindle cells [37,38], so we used LANA as a marker to monitor the KSHV-positive cells in KS tissues. As expected, no LANA staining was observed in normal skin tissues. NDRG1 is usually expressed in the basal cells of the epidermis but not in the cells of the dermis. In contrast to normal skin tissues, there were strong signals for NDRG1 in the dermal cells of the KS tissues, which also stained positive for LANA (Fig 1D). To further confirm this result, we performed a double staining assay for both NDRG1 and LANA in the same KS tissue sample section. NDRG1 staining was observed in cells that stained positive for LANA (Fig 1E). These results strongly suggest that the elevated expression of NDRG1 in KSHV-positive cells is correlated with KSHV infection and that NDRG1 may play a critical role in the persistence of viral latency.

### NDRG1 expression is upregulated by KSHV infection

**Fig 2:**
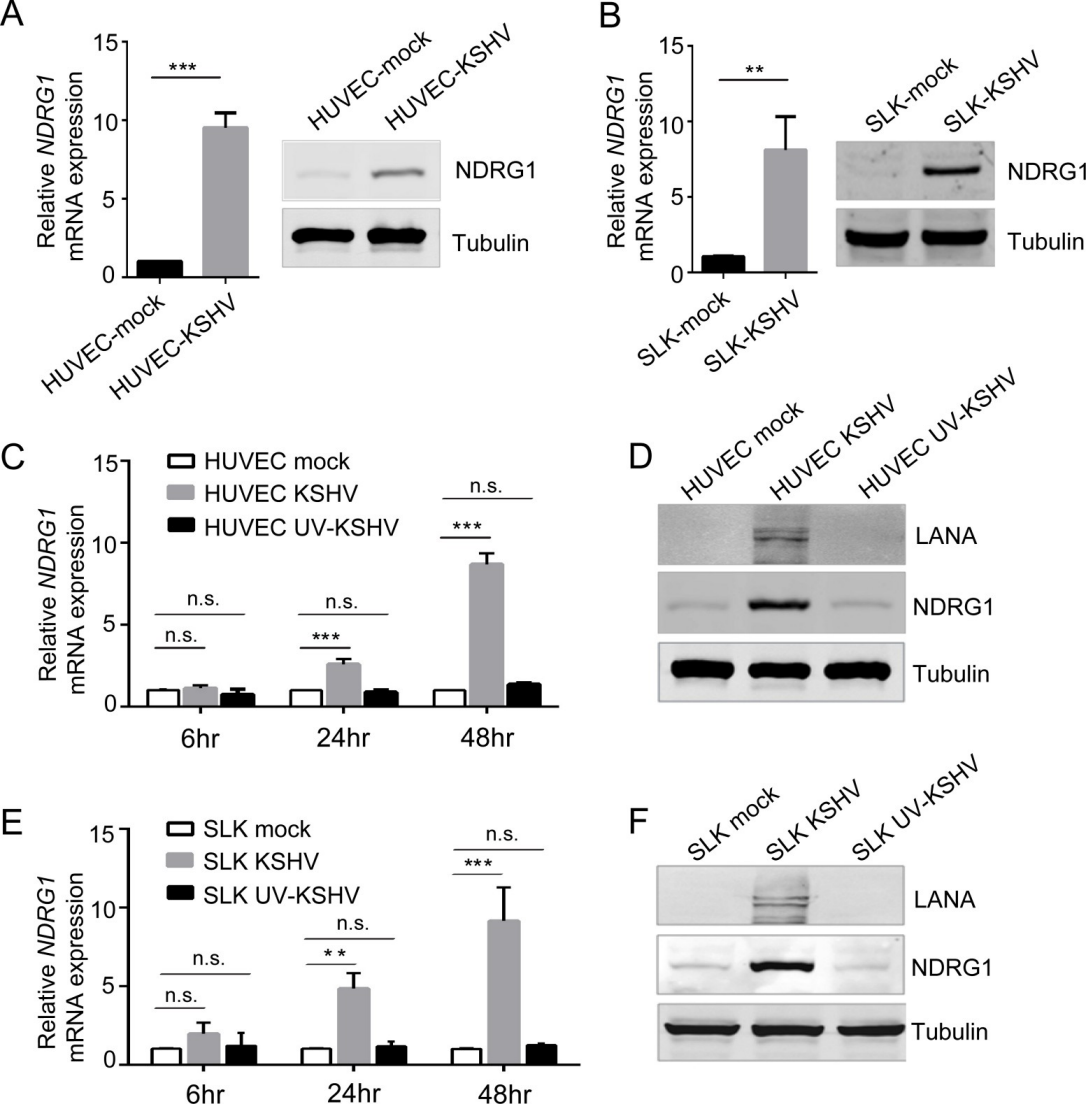
NDRG1 is up-regulated by KSHV infection. (A) HUVECs were infected with or without KSHV virions (MOI,10), and were collected at 48 hpi. The RNA and protein expression levels of NDRG1 were monitored by qPCR (left) and western blotting (right). (B) SLK cells were infected with or without KSHV virions (MOI, 5), and cells were subjected to qPCR (left) and western blotting (right) at 48 hpi. The efficiency of infection of HUVEC and SLK with KSHV were shown in S2 Fig. (C and D) HUVECs were infected with or without alive KSHV and UV-inactivated KSHV virions (MOI, 10), and were collected at 6, 24, and 48 hpi. The RNA levels of NDRG1 were detected by qPCR (C) and the protein levels at 48 hpi were examined by western blotting (D). LANA protein is 1162 amino acid in length and 220–230 kDa in size. Doublet bands of LANA in western blots are expected. (E and F) SLKs were infected with or without KSHV and UV-KSHV virions (MOI, 5), and were collected at 6, 24, and 48 hpi. The RNA levels of NDRG1 were detected by qPCR (E) and the protein levels at 48 hpi were examined by western blotting (F). qPCR data were normalized to endogenous GAPDH expression and were shown by the fold change compared to mock. Data were shown as mean ± SD, n = 3. **p<0.01, ***p<0.001.

Although NDRG1 is highly expressed in KSHV-positive cells, whether the expression of this protein is induced by KSHV infection is not clear. To explore this effect, primary human umbilical vein endothelial cells (HUVECs) were infected *de novo* with KSHV virions, and the expression of NDRG1 was examined 48 hr post infection (hpi). We found that the RNA level of NDRG1 in the KSHV-infected group was upregulated approximately 10-fold compared to that in the mock group (Fig 2A). The protein level of NDRG1 in KSHV-infected HUVECs was markedly increased, while NDRG1 protein was slightly or barely detectable in the mock HUVECs (Fig 2A), indicating that KSHV infection leads to substantial enhancement of NDRG1 expression in cells. To confirm these results, SLK cells were infected *de novo* with KSHV virions under similar conditions. Similarly, distinct augmentation of NDRG1 expression at both the RNA and protein levels was observed in KSHV-infected SLK cells compared to mock SLK cells (Fig 2B). These results confirmed that KSHV infection significantly upregulates NDRG1 expression in cells.

To further ascertain whether KSHV-induced NDRG1 was dependent upon KSHV gene expression rather than KSHV virion proteins or only the cellular stress response, HUVECs were subjected to infectious KSHV virions or UV-inactivated virions. Previous studies have demonstrated that UV-inactivated KSHV virion particles do not lessen the ability of the virions to bind or enter host cells, but the virions do so without viral gene expression [42–44]. Here, we assessed the RNA levels of NDRG1 in cells 6, 24, 48 hpi via qPCR. As shown in Fig 2C, a gradual increase in NDRG1 mRNA levels was observed in the live KSHV-infected group, while barely and changes in NDRG1 mRNA expression were observed in the UV-inactivated KSHV group. We also measured the protein levels of NDRG1 in cells 48 hpi. Consistent with the RNA levels observed, the NDRG1 protein could be detected in live KSHV-infected cells but was barely expressed in UV-inactivated KSHV-infected cells or mock cells (Fig 2D). The efficiency of infection of cells with KSHV is shown in S2 Fig and the protein level of LANA could be detected in KSHV-infected cells (Fig 2D), indicating KSHV *de nove* infected successfully. These data suggest that KSHV viral gene expression activates the expression of NDRG1.

To further confirm this hypothesis, SLK cells were also infected *de novo* with UV-inactivated KSHV virions and live KSHV virions under similar conditions. The results were similar to those obtained in HUVECs. The RNA levels of NDRG1 in SLK cells could be activated and increased by KSHV infection, while the levels remained unchanged in UV-inactivated KSHV-infected cells (Fig 2E). The protein levels of NDRG1 in SLK cells infected with UV-inactivated KSHV and live KSHV at 48 hpi were determined, and the results showed again that high expression signals could be detected in only KSHV-infected cells, not UV-inactivated KSHV-infected cells (Fig 2F). All these data indicated that KSHV viral gene expression, rather than virion proteins or the cellular stress response, activates the expression of NDRG1 during KSHV infection.

### LANA is essential for upregulation of NDRG1 expression in KSHV-infected cells

**Fig 3:**
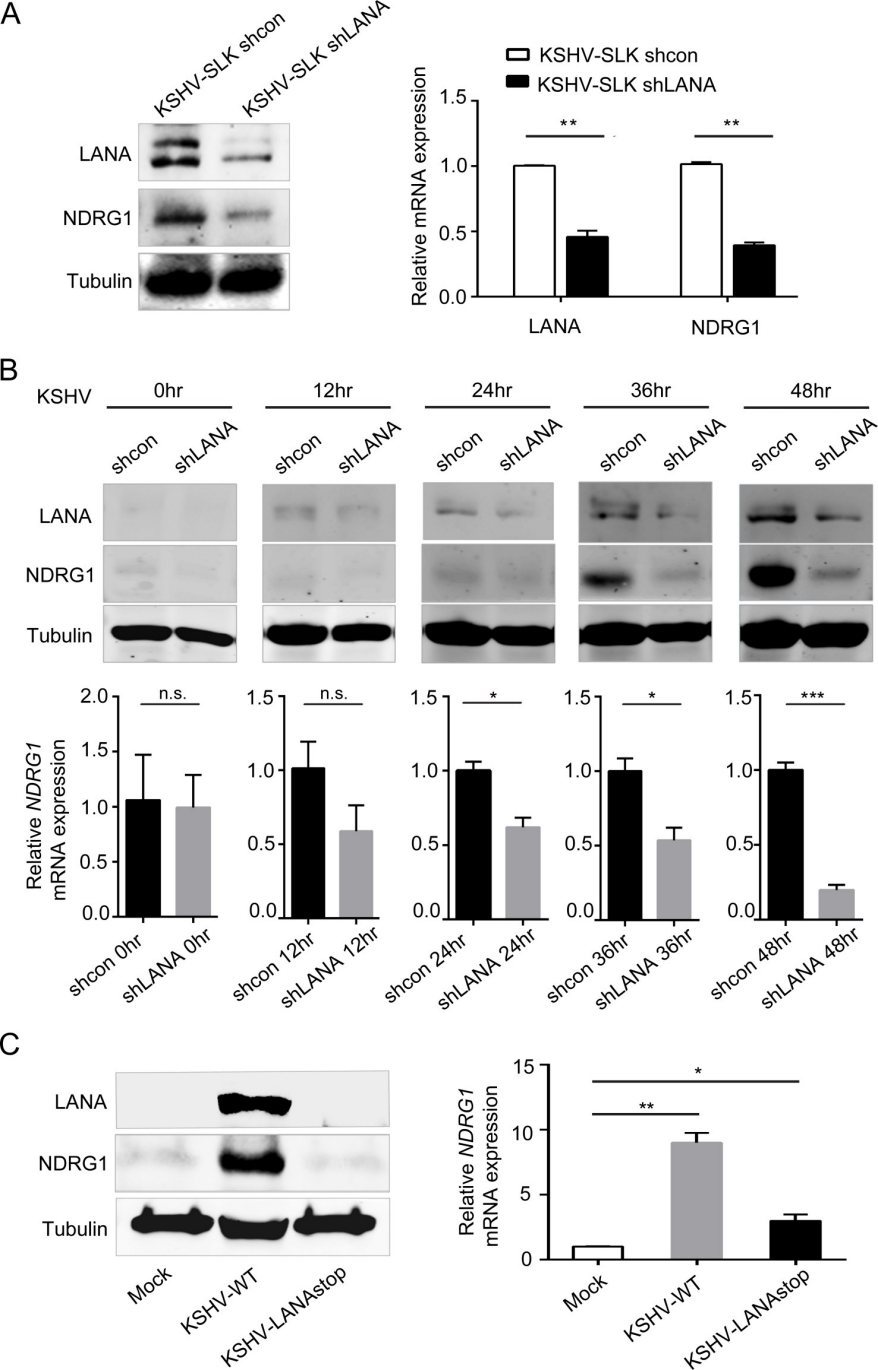
LANA is essential for upregulation of NDRG1 in KSHV infected cells. (A) KSHV-infected SLK cells were stably transduced with lentiviruses containing a LANA RNA interference plasmid (shLANA) or a vector plasmid (shcon) and were named KSHV-SLK-shcon and KSHV-SLK-shLANA, respectively. The expression of NDRG1 and LANA were detected by western blotting (left) and qPCR (right). (B) SLK cells were stably transduced with lentiviruses containing a shLANA plasmid or a shcon vector plasmid and were named SLK-shcon and SLK-shLANA, respectively. SLK-shcon and SLK-shLANA cells were infected with purified KSHV virus (MOI,5) and the efficiency of infection of cells were shown in S4 Fig. The expression of NDRG1 and LANA at 0, 12, 24, 36, and 48 hr post-infection were detected by western blotting (up panel) and qPCR (lower panel). (C) SLK cells were infected with or without the wild-type (WT) or the LANA-depleted (LANA-stop) KSHV (MOI, 5) and harvested at 48 hr post-infection. The protein levels of NDRG1 and LANA were measured by western blotting (left). The RNA level of NDRG1 was determined by qPCR (right). qPCR data were normalized to the level of endogenous GAPDH in each group. Data were shown as mean ± SD, n = 3, *p<0.05, **p<0.01, ***p<0.001.

The above results demonstrated that the products generated by KSHV genes regulate the expression of NDRG1. Next, we sought to identify the viral protein that regulates NDRG1 expression. The latent phase of KSHV is characterized by the expression of a limited number of viral genes. LANA is the most abundant viral protein expressed during latency and is required for various vital functions for persistence of KSHV infection [45]. Hence, we speculated that LANA might be responsible for regulating the expression of NDRG1. To test this possibility, we first investigated the role of LANA in the maintenance of NDRG1 expression. We silenced LANA in KSHV-infected SLK cells via RNA interference. Indeed, the expression of NDRG1 was greatly decreased when LANA expression was knocked down (Fig 3A), which is consistent with the result in PEL cells (S3 Fig) In addition, the NDRG1 mRNA level was significantly decreased, which was accompanied by reduction of LANA expression (Fig 3A). These data suggested that LANA plays a role in maintaining NDRG1 expression.

Next, we explored the role of LANA in the induction of NDRG1 in KSHV infection. For this purpose, we first constructed two stable SLK cell lines expressing SLK-shcon and SLK-shLANA. The purified KSHV virus was used to infect these two stable cell lines, and NDRG1 was detected at both the protein and RNA levels. The efficiency of infection of SLK-shcon and SLK-shLANA cells with KSHV were shown in S4 Fig. Gradual augmentation of NDRG1 expression was observed in the SLK-shcon group, and the peak NDRG1 level was observed at 48 hpi, along with an increase in LANA expression, during KSHV infection (Fig 3B). However, NDRG1 failed to be induced sufficiently when LANA was inhibited in SLK-shLANA cells (Fig 3B), suggesting that LANA plays a key role in inducing NDRG1 during KSHV infection.

To further validate these results, SLK cells were infected with KSHV and LANA-depleted KSHV [46] or were not infected. Similarly, NDRG1 expression was not induced at the protein level when LANA was eliminated (Fig 3C), although the RNA level of NDRG1 was slightly enhanced compared with that in uninfected cells (Fig 3C), suggesting that LANA is responsible for inducing NDRG1 during KSHV infection.

Taken together, these results demonstrate that LANA plays an essential role in the upregulation of NDRG1 expression.

### Silencing NDRG1 results in highly deficient viral episome persistence

**Fig 4:**
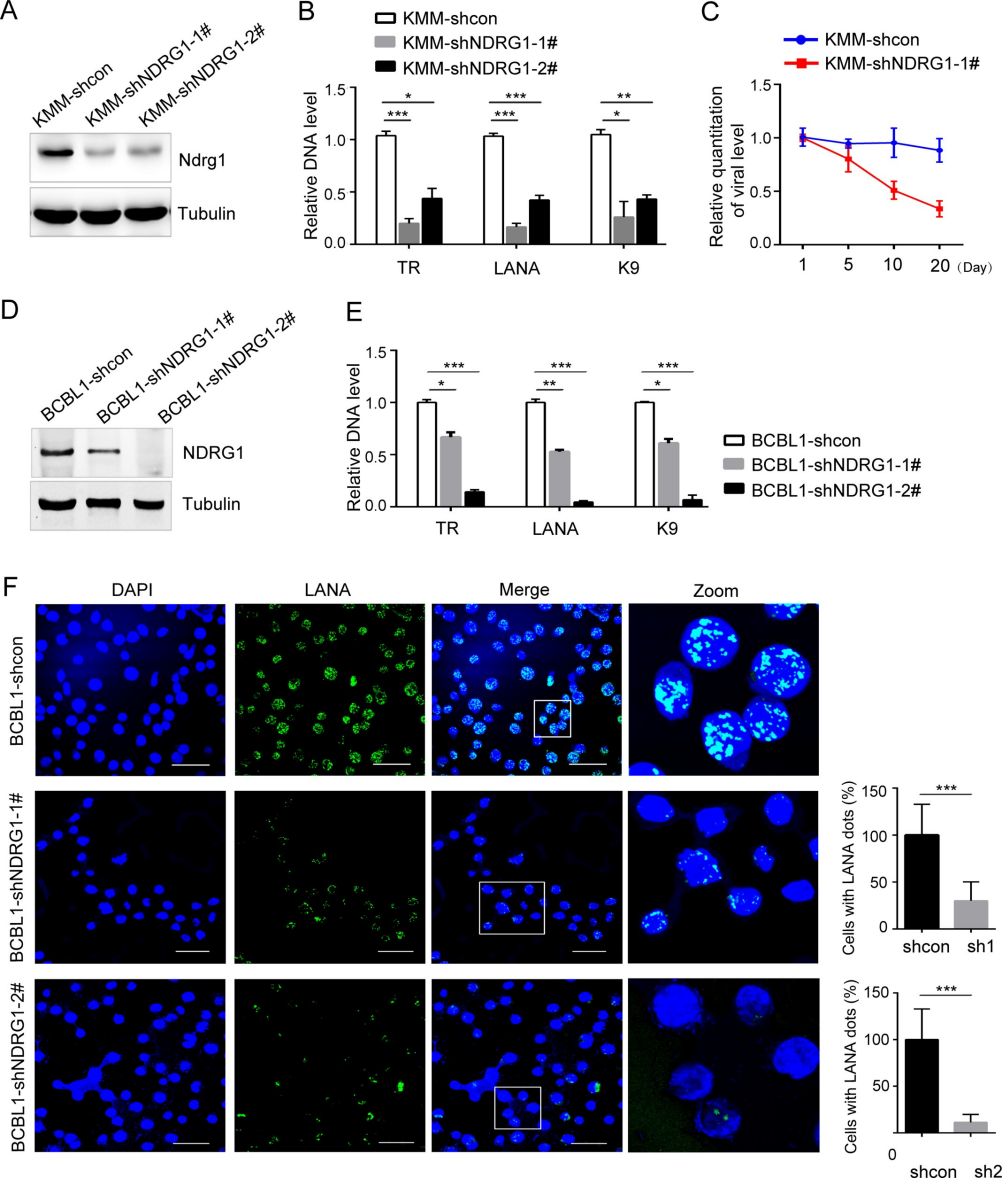
Silencing NDRG1results in highly deficient viral episome persistence. (A) The knockdown efficiency of NDRG1 in KMM-shNDRG1 cells were determined by western blotting. (B) The viral genomes in KMM-shNDRG1 and KMM-shcon cells were extracted and measured by qPCR and normalized to the copy number of the GAPDH gene. The relative viral genomic copy number in the vector group was set as 1.0. Data were shown as mean ± SD, n = 3, *p<0.05, **p<0.01, ***p<0.001. (C) Loss of KSHV genomes from KMM-shNDRG1 and KMM-shcon cells was monitored at each time point. (D) The knockdown efficiency of NDRG1 in BCBL1-shNDRG1 cells. (E) The DNA levels of viral genomes in BCBL1 cells in the presence or absence of NDRG1 detected by qPCR and normalized to the copy number of the GAPDH gene. The relative viral genomic copy number in the vector group was set as 1.0. Data were shown as mean ± SD, n = 3, *p<0.05, **p<0.01, ***p<0.001. (F) LANA immunostaining (green) of BCBL1 cells in the absence or presence of NDRG1 knockdown. DAPI (blue) stains DNA in cell nuclei. Scale bars represent 25μm. Insets show the enlarged images of the boxed areas. Quantification of cells containing LANA dots (right). Data were shown as mean ± SD, n = 18, ***p<0.001.

To explore the role of NDRG1 in KSHV infection, we knocked down endogenous NDRG1 in KMM cells. KMM cells were transduced with a lentivirus containing two specific shRNAs (sh-NDRG1-1# and sh-NDRG1-2#) and a control shRNA (sh-control), followed by selection. The NDRG1 expression were greatly decreased in KMM-shNDRG1-1# and 2# compared with KMM-shcon (Fig 4A).

Because the previous data indicated that NDRG1 may play a critical role in the persistence of viral latency, we hypothesizes that the expression of viral genes might be changed in the absence of NDRG1 expression. We checked the transcriptional levels of a representative latent gene (LANA) and a lytic gene (RTA) in KMM-shNDRG1 cells. Surprisingly, both LANA and RTA levels were significantly diminished upon the reduction of NDRG1 levels in KMM cells (S5 Fig), indicating that NDRG1 might regulate the KSHV DNA levels, thereby simultaneously affecting both latent and lytic gene expression at the RNA level. To validate this hypothesis, we assessed the DNA levels of the viral genomes in KMM cells approximately 20 days after NDRG1depletion in KMM cells by qPCR. The results showed that the DNA levels of TR, LANA, and K9 in KMM-shNDRG1 cells were markedly lower than those in KMM-shcon cells (Fig 4B). We further assessed the DNA levels of the viral genomes at days 1, 5, 10, and 20 after NDRG1 knockdown in KMM cells. There was a distinct loss of KSHV episomal genomes in the absence of NDRG1, whereas KSHV episome levels remained relatively stable in the control cells (Fig 4C). To obtain direct evidence, we performed fluorescence *in situ* hybridization (FISH) analysis. Indeed, the FISH results showed that the levels of TR DNA, a region on the KSHV viral genome, were decreased in the NDRG1 knockdown KMM-shNDRG1 cells (S6 Fig). These findings suggested that silencing NDRG1 hampers viral genome persistence in cells latently infected with KSHV.

To further verify this phenomenon, we examined the effect of NDRG1 downregulation on the maintenance of viral episomes in BCBL1, which is a PEL cell line latently infected with KSHV. We constructed stably transfected BCBL1 cell lines, including BCBL1-shcon, BCBL1-shNDRG1-1#, and BCBL1-shNDRG1-2#. NDRG1 expression in BCBL1-shNDRG1 cells was successfully reduced (Fig 4D). Indeed, the DNA levels of the intracellular viral genomes were decreased in BCBL1-shNDRG1 cells, which were tested approximately 20 days after NDRG1depletion in cells (Fig 4E). As LANA colocalizes with KSHV episomes on chromosomes in PEL cells [6], we also detected LANA by immunostaining to determine the changes in viral episomes. In NDRG1 knockdown cells, the LANA dots were significantly reduced, and the percentage of cells containing LANA dots was approximately 30% in BCBL1-NDRG1-1# and approximately 12% in BCBL1-NDRG1-2# compared with BCLB1-shcon cells (Fig 4F). Taken together, these results demonstrated that inhibition of NDRG1 in KSHV-infected cells results in greatly decreased persistence of the KSHV genome and suggested that NDRG1 plays a role in the maintenance of the viral genome in KSHV-infected cells.

### NDRG1 directly interacts with the DNA polymerase clamp PCNA

**Fig 5:**
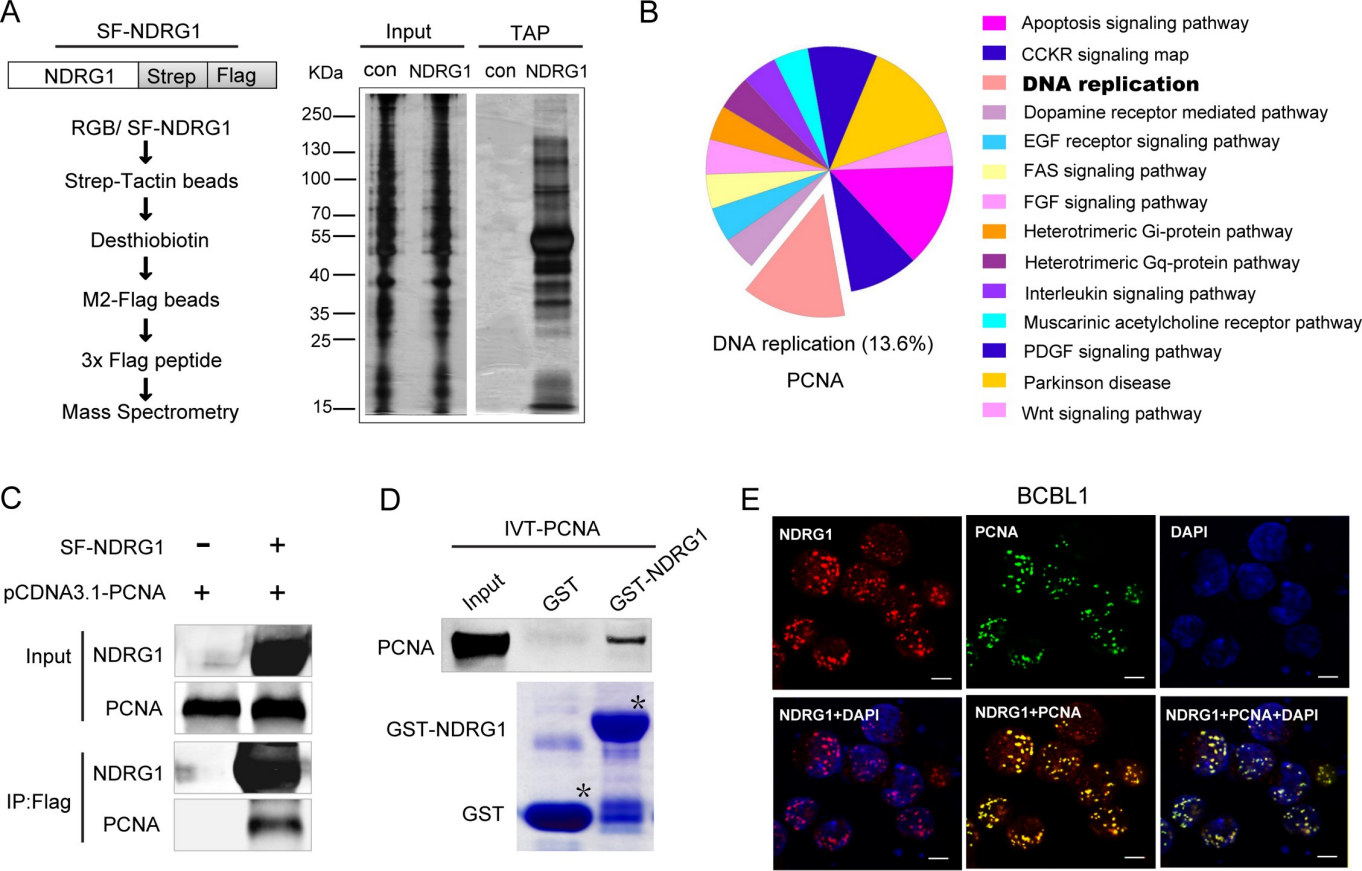
NDRG1 directly interacts with PCNA. (A)Schematic procedure for purification and identification of NDRG1 binding proteins via TAP assay (left). Plasmid expressing Strep-Flag-tagged NDRG1 was stable transfected into KSHV positive iSLK.RGB cells. The equivalent empty vector was stable transfected as a control. Cell lysates were subjected to affinity purification with streptavidin beads, followed by IP with flag M2 beads. The purified elutes were resolved by SDS-PAGE and visualized with silver staining (right), and were also analyzed by MS. (B) Pathway pie chart showing classified and predicted functions of MS identified proteins. (C) Co-IP of NDRG1 and PCNA in HEK293T cells. Strep-Flag-tagged NDRG1 was transfected into cells along with pCDNA3.1-PCNA or empty vector controls. After affinity purification with M2-Flag beads, the purified proteins along with input samples were detected by western blotting with anti-NDRG1 and anti-PCNA antibodies. (D) *In vitro* interaction between NDRG1 and PCNA via GST pull down assay. Purified GST, and GST-fused full length NDRG1 were subjected to SDS-PAGE and Coomassie Blue staining (lower panel). Purified beads were incubated with equivalent *in vitro* translated IVT–PCNA, and pulled down proteins were subjected to western blotting detection (upper panel). (E) NDRG1 colocalized with PCNA in the nucleus. Cells were fixed and probed with rabbit antibody against NDRG1 and mouse antibody against PCNA, followed by incubation with goat anti-rabbit IgG conjugated with Alexa Fluor 555 (red), goat anti-mouse IgG conjugated with Alexa Fluor 488 (green), DAPI (blue). Scale bars represent 5μm.

To explore the potential mechanism by which NDRG1 regulates the persistence of viral episomes during KSHV latency, we performed tandem affinity purification/mass spectrometry (TAP-MS) to identify proteins that interact with NDRG1. Strep-FLAG (SF)-tagged NDRG1 was expressed in KSHV-positive iSLK.RGB cells. Then, the cell lysates were subjected to affinity purification with streptavidin beads, followed by immunoprecipitation (IP) with FLAG M2 beads (Fig 5A). The purified eluates were analyzed by MS. Several nucleoproteins, shown in S4 Table, were identified by peptide correlation with the International Protein Index database, suggesting that NDRG1 may play important roles in cell nuclei. The classified and predicted functions of the identified proteins are shown in the pathway pie chart in Fig 5B, which was created using the PANTHER system (http://www.pantherdb.org/). Interestingly, there were approximately 13.6% potential NDRG1-binding proteins related to DNA replication, which would influence the viral copy numbers in KSHV-infected cells. PCNA, among these proteins, is a replication processivity factor and functions as a DNA polymerase clamp that is essential for DNA replication [29,30]. We speculated that NDRG1 might interact with PCNA and be involved in regulating viral DNA replication, thereby contributing to the maintenance of viral copy numbers in KSHV-infected cells.

To verify this hypothesis, we performed a co-IP assay, and the results showed that NDRG1 interacted with PCNA (Fig 5C). To further determine whether this interaction was direct, we investigated the interaction using an *in vitro* binding assay. As shown in Fig 5D, IVT-PCNA (*in vitro* translated PCNA) was directly bound to full-length NDRG1 (1–394 aa). To support the results, we also performed an immunofluorescence (IF) analysis to assess the localization of endogenous PCNA and NDRG1 in KSHV-positive BCBL1 cells. The results showed that NDRG1 colocalized with PCNA in the nuclei of BCBL1 cells (Fig 5E). Taken together, these results demonstrate that NDRG1 directly interacts with PCNA *in vitro* and *in vivo*, suggesting that NDRG1 might play a role in DNA replication.

### NDRG1 forms a complex with LANA and PCNA

**Fig 6:**
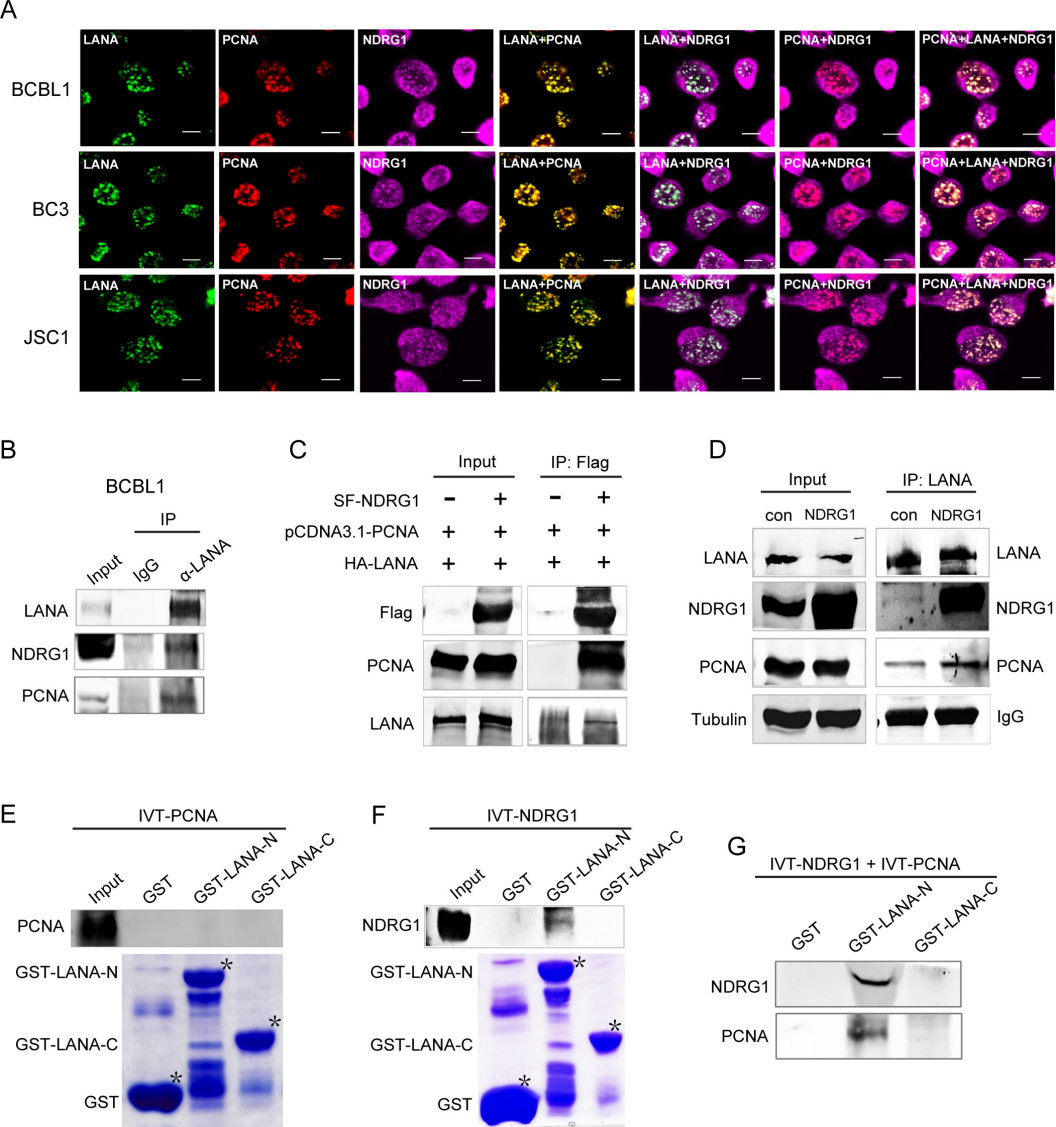
NDRG1 forms a complex with LANA and PCNA. (A) LANA colocalized with NDRG1 and PCNA in the nucleus. Cells were fixed and probed with rat antibody against LANA, mouse antibody against PCNA, and rabbit antibody against NDRG1, followed by incubation with goat anti-rat IgG conjugated with Alexa Fluor 488 (green), goat anti-mouse IgG conjugated with Alexa Fluor 555 (red), goat anti-rabbit IgG conjugated with Alexa Fluor 680 (purple). Scale bars represent 5μm. (B) Co-IP of endogenous LANA, NDRG1, and PCNA in BCBL1 cells. Cell lysates were subjected to IP with anti-LANA mouse monoclonal antibody(1B5) or mouse IgG controls. Purified proteins along with input samples were detected by western blotting with anti-LANA, anti-NDRG1, and anti-PCNA antibodies. (C) Co-IP of LANA, NDRG1, and PCNA in HEK293T cells. Strep-Flag-tagged NDRG1 was transfected into cells along with pCDNA3.1-PCNA and HA-LANA or empty vector controls. After affinity purification with M2-Flag beads, the purified proteins along with input samples were detected by western blotting with anti-LANA, anti-NDRG1 and anti-PCNA antibodies. (D) Co-IP of LANA, NDRG1, and PCNA in HEK293T-Strep-Flag-LANA cells. pCDNA3.1-NDRG1 or pCDNA3.1-vector was transfected into cells. After affinity purification with M2-Flag beads, the purified proteins along with input samples were detected by western blotting with anti-LANA, anti-NDRG1 and anti-PCNA antibodies. Tubulin was performed as the loading control of input samples, and IgG was used as the loading control of M2-Flag beads. (E) *In vitro* interaction between LANA and PCNA via GST pull down assay. Purified GST, and GST-fused LANA-N (1–340 aa) and GST-fused LANA-C (1022–1162 aa) beads were subjected to SDS-PAGE and Coomassie Blue staining (lower panel). Purified beads were incubated with equivalent *in vitro* translated IVT–PCNA, and pulled down proteins were subjected to western blotting detection (upper panel). (F) *In vitro* interaction between LANA and NDRG1 via GST pull down assay. Purified GST, and GST-fused LANA-N and GST-fused LANA-C beads were subjected to SDS-PAGE and Coomassie Blue staining (lower panel). Purified beads were incubated with equivalent IVT–NDRG1, and pulled down proteins were subjected to western blotting detection (upper panel). (G) *In vitro* interaction between LANA and PCNA in the presence of NDRG1 via GST pull down assay. Purified GST, and GST-fused LANA-N and GST-fused LANA-C beads were incubated with equivalent IVT-PCNA and IVT–NDRG1, and pulled down proteins were subjected to western blotting detection.

PCNA can be loaded onto the KSHV TR region by LANA indirectly, and this loading is a rate-limiting step in viral DNA replication [23,27]. Based on the above data, we hypothesized that NDRG1 might form a complex with LANA and PCNA and regulate the replication of KSHV episomal DNA. To determine whether NDRG1 forms a complex with LANA and PCNA in cells naturally infected with KSHV, we performed an IF assay in PEL cells. We found that NDRG1 colocalized with PCNA and LANA in the nuclei of BCBL1, BC3, and JSC1 cells (Fig 6A). We also performed a co-IP assay in BCBL1 cells. The results showed that endogenous LANA could interact with PCNA and NDRG1 in cells naturally infected with KSHV (Fig 6B). Previous studies have been reported that LANA can bind TR region of episomes itself [16] and PCNA can be recruited onto TR region [23,27]. In order to exclude the possibility that NDRG1 and PCNA are associated with KSHV episomes but not because of a meaningful complex with LANA, we also preformed an anti-CTCF IP as a control, which is an antibody against a KSHV episome-associated antigen that does not involve targeting LANA [47–49]. The results showed that NDGR1 and PCNA are enriched in an anti-LANA pulldown in BCBL1 cells, compared to an anti-CTCF pulldown, indicating that endogenous LANA-specific associates with NDRG1 and PCNA (S7 Fig).

To further confirm this hypothesis, we also performed a co-IP assay in HEK293T cells. The results showed that NDRG1 coimmunoprecipitated with PCNA and LANA (Fig 6C). Next, we altered the amount of NDRG1 while keeping the amount of LANA and PCNA unchanged to check whether the quantity of PCNA that coimmunoprecipitated with LANA would vary. For this purpose, we first generated a stably transfected HEK293T-Strep-Flag-LANA cell line that constantly expressed SF-LANA, endogenous NDRG1 and PCNA. Then, a NDRG1-containing plasmid was transfected into the HEK293T-SF-LANA cells to upregulate NDRG1 expression in the cells. The results showed that the quantity of PCNA that coimmunoprecipitated with LANA increased when the amount of NDRG1 was increased (Fig 6D). These data indicated that NDRG1 forms a complex with LANA and PCNA and that NDRG1 mediates the amount of PCNA recruited by LANA.

Because LANA cannot directly bind to PCNA [23,27], we speculated that NDRG1 acts as an adaptor, connecting LANA and PCNA. First, we performed an *in vitro* binding assay to test whether LANA binds to PCNA. As shown in Fig 6E, LANA did not directly bind to IVT-PCNA, which is consistent with previous reports [23,27]. Next, we performed an *in vitro* binding assay to test whether LANA binds to NDRG1. The results showed that *in vitro* translated NDRG1 (IVT-NDRG1) directly bound to the N-terminal domain of GST-fused LANA instead of the C-terminal domain (Fig 6F). To test whether LANA would bind PCNA *in vitro* in the presence of NDRG1, IVT-PCNA and IVT-NDRG1 were incubated with purified GST-fused LANA beads. The results showed that LANA could bind PCNA in the presence of NDRG1 (Fig 6G), suggesting that NDRG1 bridges the interaction between LANA and PCNA. Taken together, these data demonstrate that NDRG1 forms a complex with LANA and PCNA *in vivo* and *in vitro*.

### NDRG1 mediates the recruitment of PCNA by LANA onto the KSHV genome

**Fig 7:**
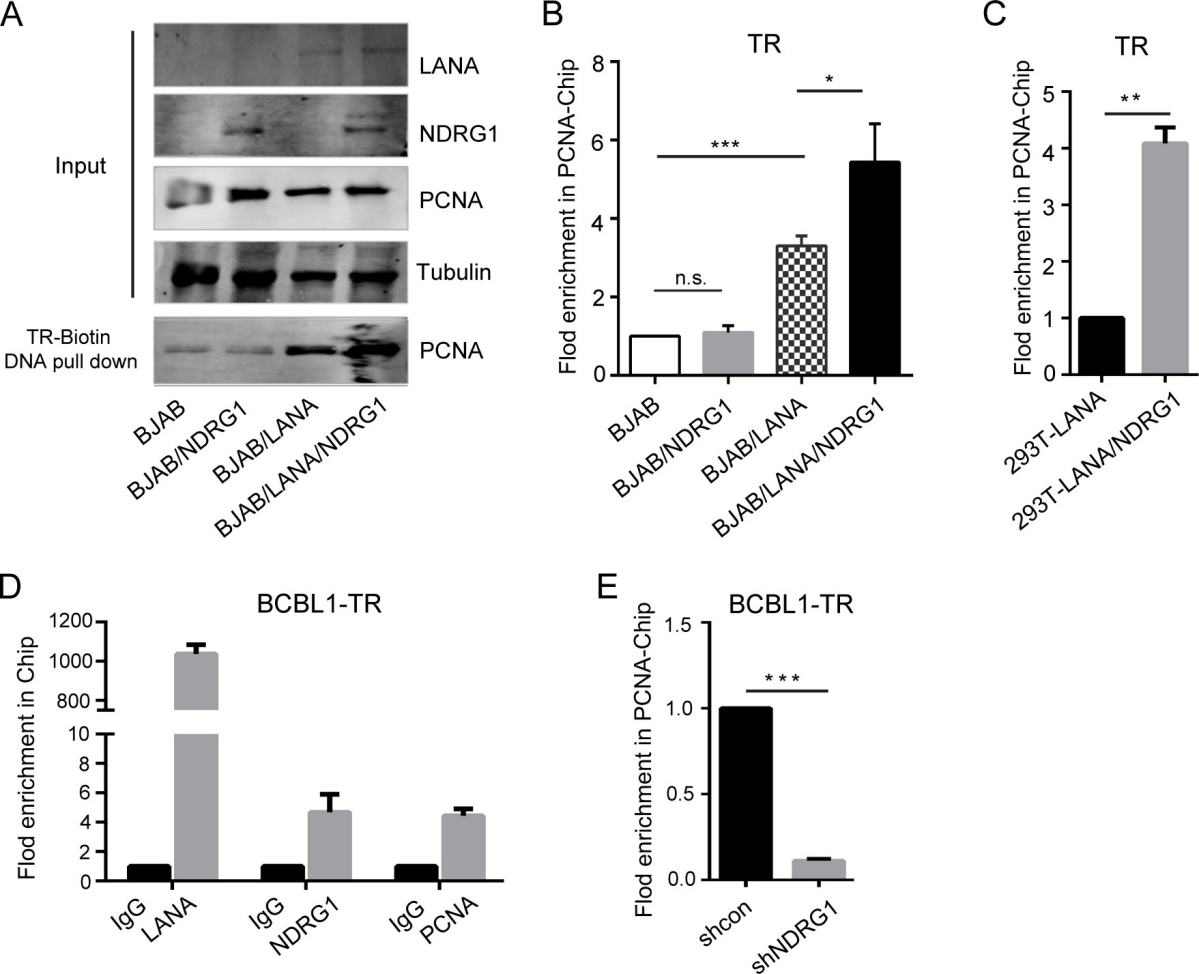
NDRG1 mediates the recruitment of PCNA by LANA onto the KSHV genome. (A) *In vitro* TR biotin-labeled DNA pull-down assay. NDRG1 and/or LANA was transfected into BJAB cells. After 24 hr, cells were lysed and five percent of the cell lysates were kept as inputs, and the remainder was incubated with purified biotin-TR DNA fragment and immobilized to streptavidin beads. The inputs and the pulled down products were analyzed by western blotting. The full-length western blot images for the antibodies and molecular weight markers of In vitro TR biotin-labeled DNA pull-down assay is shown in S8 Fig. (B) ChIP assay for PCNA binding to TR DNA after transfection of p8TR into BJAB, BJAB/NDRG1, BJAB/LANA, or BJAB/LANA/NDRG1 cells. (C) ChIP assay for PCNA binding to TR DNA after transfection of p8TR into HEK293T-LANA or HEK293T-LANA/NDRG1 cells. (D) ChIP assay for LANA, NDRG1,or PCNA bound to TR DNA of KSHV genome in BCBL1 cells. (E) BCBL1-shcon and BCBL1-shNDRG1 cells were harvested and subjected to ChIP with anti-PCNA antibodies. Data were shown as mean ± SD, n = 3, *p<0.05, **p<0.01.

Based on the above results, we speculated that NDRG1 may mediate the recruitment of PCNA by LANA onto the TR DNA and facilitate viral DNA replication. To test this hypothesis, we performed an *in vitro* pull-down assay by using TR biotin-labeled DNA. NDRG1 and/or LANA were transfected into BJAB cells, which express endogenous PCNA but lack NDRG1 and LANA. Twenty-four hours later, the cells were lysed, incubated with the biotin-TR DNA fragment, and immobilized to streptavidin beads. The inputs and the pull-down products were analyzed by western blotting. As shown in Fig 7A, the presence of NDRG1 in BJAB did not alter the levels of PCNA and LANA. In such cases, we found that the amount of PCNA bound with TR did not change when NDRG1 was present but LANA was absent. Consistent with previous reports [23,27], the loading of PCNA onto DNA was dramatically enhanced in the presence of LANA (Fig 7A). In addition, NDRG1 was capable of enhancing the loading of PCNA onto TR in the presence of LANA (Fig 7A). The full-length western blot images of Fig 7A cutoff bands are shown in S8 Fig. These results indicated that the enhancement of PCNA enrichment on TR by NDRG1 is dependent on LANA-mediated recruitment of PCNA onto the TR DNA.

We further assessed whether NDRG1 mediates the recruitment of PCNA to TR DNA by LANA in cells by a chromatin immunoprecipitation (ChIP) assay. NDRG1 and/or LANA were transfected into BJAB cells along with p8TR plasmids. The cells were lysed for the PCNA-ChIP assay 24 hr post transfection. As shown in Fig 7B, PCNA was substantially enriched at the TR DNA in the presence of LANA. The enrichment of PCNA at the TR was predicted to be further enhanced in the presence of both NDRG1 and LANA. To confirm this hypothesis, we performed a PCNA-ChIP assay in HEK293T-LANA cells. Similarly, the results showed that the enrichment of PCNA at the TR was enhanced at high NDRG1 levels in the cells (Fig 7C).

To determine the role of NDRG1 in the recruitment of PCNA to the KSHV genome, we first performed ChIP to assay the presence of LANA, NDRG1, and PCNA at the TR DNA in KSHV-infected BCBL-1 cells. As expected, LANA and PCNA were enriched at the TR DNA, as previously reported [23,27]. In addition, we also found that NDRG1 was enriched at the TR DNA, suggesting that NDRG1 may play a role in the KSHV genome (Fig 7D). To determine whether NDRG1 mediates the recruitment of PCNA to the KSHV genome, we performed a PCNA-ChIP assay in BCBL1-shcon and BCBL1-shNDRG1 cells. As shown in Fig 7E, the level of PCNA enrichment to the KSHV genome was hampered when NDRG1 was knocked down, suggesting that NDRG1 mediates the recruitment of PCNA to the KSHV genome by LANA.

### NDRG1 is crucial for LANA-mediated DNA replication and episome persistence

**Fig 8:**
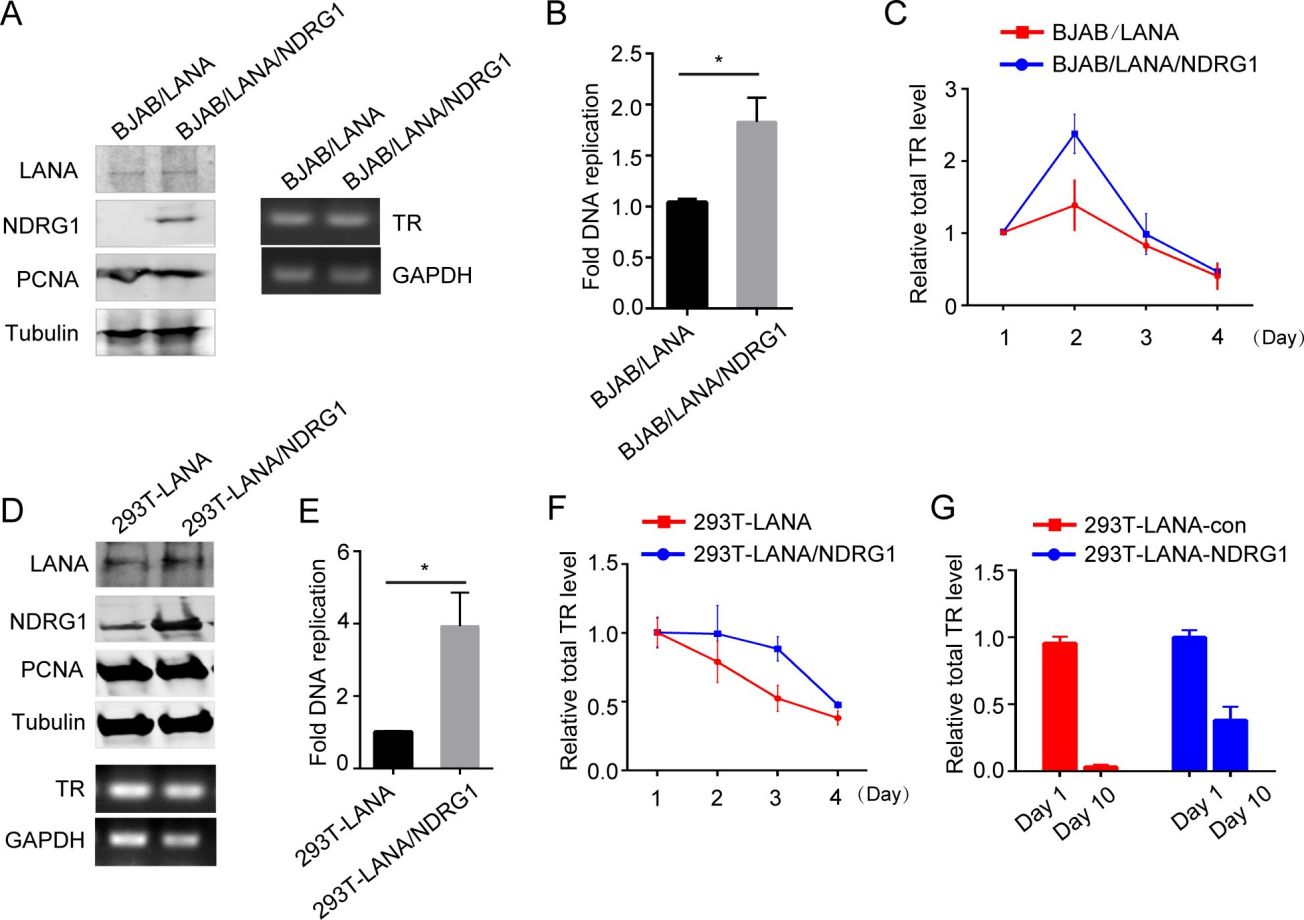
NDRG1 is crucial for LANA-mediated DNA replication and episome persistence. (A) The p8TR plasmid was transfected into BJAB cells along with LANA and NDRG1 or vector plasmids. Cells were collected at 24 hr post-transfection. Western blotting analysis for LANA, NDRG1, PCNA, and Tubulin were performed (left). The amount of p8TR DNA were analyzed by PCR amplification and DNA gel analysis (right). (B) Cells were collected at 72hr post-transfection. Hirt’s DNA from cells were purified and followed by *DpnI* and *ExoIII* digestion. LANA-mediated TR DNA replication were determined by qPCR. Data were shown as mean ± SD, n = 3, *p<0.05. (C) Cells were collected at day1,2,3,4 post-transfection. Total TR DNA level were analyzed by qPCR. (D) The p8TR plasmid was transfected into HEK293T-LANA cells along with NDRG1 or vector plasmids. Cells were collected at 24hr post-transfection. Western blotting analysis for LANA, NDRG1, PCNA, and Tubulin were performed (upper). The amount of p8TR DNA were analyzed by PCR amplification and DNA gel analysis (lower). (E) Cells were collected at 72hr post-transfection. Hirt’s DNA from cells were purified and followed by *DpnI* and *ExoIII* digestion. LANA-mediated TR DNA replication were determined by qPCR. Data were shown as mean ± SD, n = 3, *p<0.05. (F) Cells were collected at day1,2,3,4 post-transfection. Total TR DNA level were analyzed by qPCR. (G) qPCR analysis assessing the presence of TR DNA in HEK293T-LANA cells with or without NDRG1 after 10 days in the absent of drug selection.

To assess the role of NDRG1 in LANA-mediated DNA replication, we performed a LANA-mediated DNA replication assay [23,50]. The p8TR plasmid was transfected into BJAB cells along with LANA and NDRG1 or vector plasmids. First, to ensure the efficiency of transfection, we collected cells at 24 hr post transfection and assayed the expression of LANA, NDRG1, and PCNA by western blotting (Fig 8A left) and the amount of p8TR DNA by qPCR and DNA gel analysis (Fig 8A right). These results indicated similar transfection efficiencies of the LANA and TR plasmids in both groups of BJAB cells. Next, we collected cells at 72 hr post transfection and determined the effects of NDRG1 on LANA replication. As shown in Fig 8B, DNA replication activity was significantly enhanced in the cells expressing NDRG1 compared with those lacking NDRG1, indicating that NDRG1 assists LANA-mediated DNA replication.

The process of TR DNA replication affects the maintenance of viral episomes in dividing cells. To further assess the role of NDRG1 in DNA replication and maintenance of viral episomes, we measured the short-term retention rates of TR plasmid levels post transfection in the absence of selective agents. The p8TR plasmid was transfected into BJAB cells along with LANA and NDRG1 or vector plasmids, and the total TR level was measured at days 1, 2, 3, and 4 post transfection by qPCR. Consistent with previous reports [51], the total TR plasmid levels in the cells remained stable for 48 hr post transfection and gradually decreased with the reduction of transfected LANA (Fig 8C). Interestingly, NDRG1 enhanced the total levels of TR plasmids maintained in the cells, especially at 48 hr, and the levels then gradually decreased. These results indicated that NDRG1 plays a role in DNA replication and maintenance of viral episomes.

To further confirm these results, the p8TR plasmids along with NDRG1 or vector plasmids were transfected into HEK293T-LANA cells stably expressing LANA and low levels of endogenous NDRG1. The results in Fig 8D show the efficiency of transfection. Cells were collected at 72 hr post transfection, and the effects of NDRG1 on LANA-mediated DNA replication were determined. Consistent with the above results, DNA replication activity was significantly enhanced in the cells expressing high levels of NDRG1 (Fig 8E). Short-term retention of TR plasmids post transfected in the absence of selective agents was also measured. The results showed that NDRG1 further reduced the loss of TR plasmids (Fig 8F), indicating that NDRG1 plays a role in DNA replication and may also have an effect on TR maintenance.

To test whether NDRG1 assists LANA in long-term retention of TR, the p8TR plasmid was transfected into HEK293T-LANA cells along with NDRG1 or vector plasmids. Because TR plasmids can be maintained long term in subpopulations in the presence of LANA without drug selection [51], the cells were cultured without drug selection. Considering the loss of NDRG1 plasmids in dividing cells post transfection, we replenished the NDRG1 plasmids at days 3 and 7. Higher levels of TR DNA were detected at day 10 in the presence of both LANA and NDRG1 than in the presence of LANA but absence of NDRG1 (Fig 8G), suggesting that NDRG1 plays a role in TR plasmid persistence.

## Discussion

**Fig 9:**
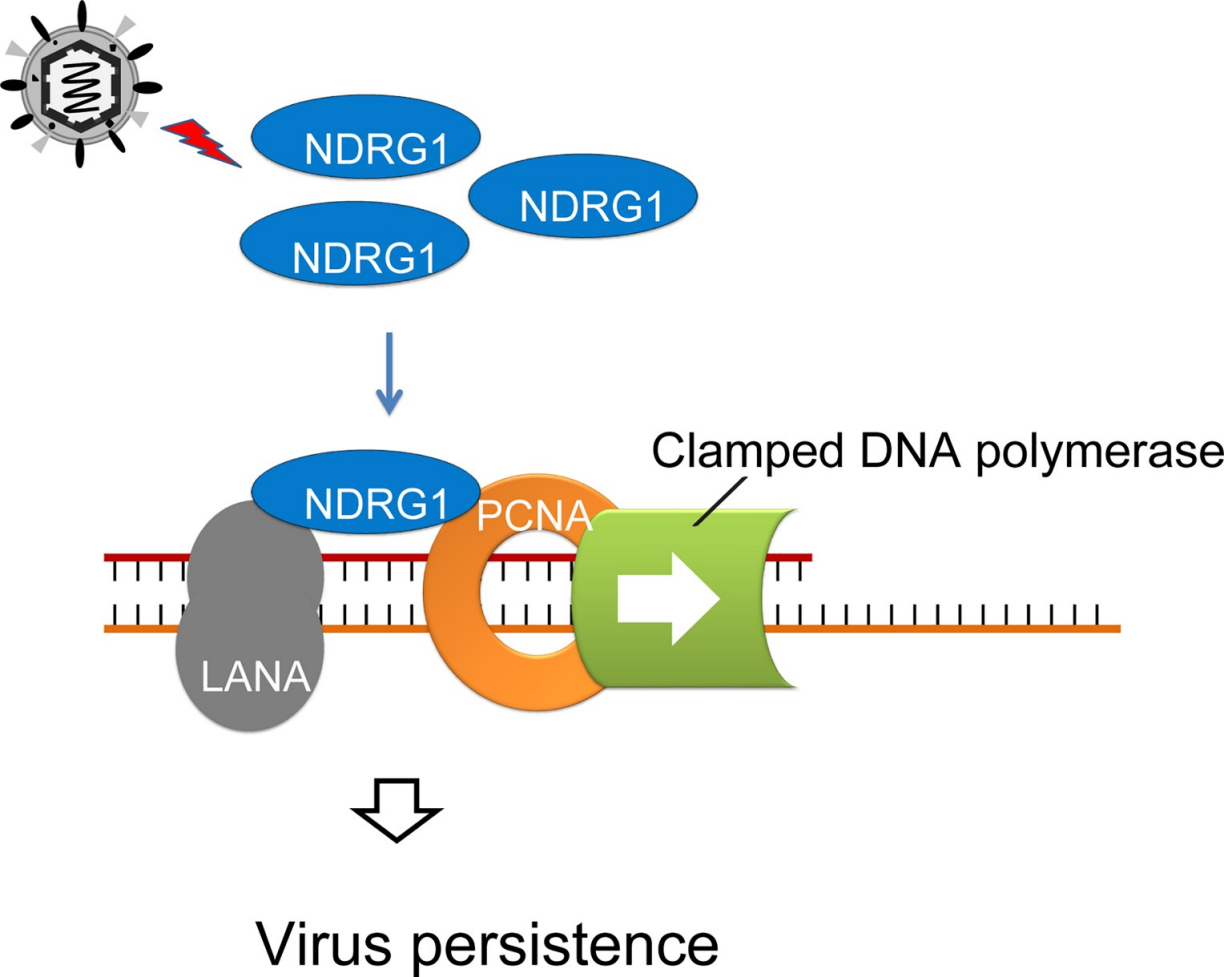
A schematic working model. Host NDRG1 is up-regulated by KSHV infection. NDRG1 functions as a scaffold protein that forms a complex with PCNA and the viral protein LANA, thereby helping LANA load PCNA onto the viral genome and facilitating the replication of the viral genome.

In cells latently infected with KSHV, the viral genome levels are constant. Similar to other gammaherpesviruses, KSHV is distinctly adept at establishing stable latent infections and maintaining a constant copy number of viral episomes in proliferating host cells throughout the life of the host. For instance, KSHV-positive PEL cells contain 50–200 copies of viral episomes per cell, and the copy number of the KSHV genome remains constant after multiple cell division events [39–41]. To maintain this stable episome in growing cells, the viral genome typically replicates once per cell cycle and is evenly distributed into daughter cells [5,6,8–13]. However, the regulation of this process is complicated. Anything that interferes with the replication of viral episomes along with cell DNA or hampers efficient distribution of replicated episomes into daughter nuclei will hinder the maintenance of viral episomes. This process involves the utilization of host machinery by KSHV; however, the underlying mechanisms have not been fully elucidated. In our study, we found that the cellular protein NDRG1 is highly upregulated by KSHV infection. Silencing of NDRG1 results in greatly decreased KSHV episome persistence. In terms of mechanism, we found that NDRG1 serves as a scaffold protein that bridges the interaction between LANA and PCNA and mediates the recruitment of PCNA to the KSHV genome by LANA. Finally, we demonstrated that NDRG1 enhances LANA-mediated replication and thus influences the persistence of the KSHV genome. The proposed working model is summarized in Fig 9.

Previously, numerous studies have shown that the host protein NDRG1 is a multifunctional protein that is involved in carcinogenesis, differentiation, stress response, immunity, etc. [33–36], but none of these studies linked this protein to viral DNA replication. Recently, some groups have reported that NDRG1 might be associated with viral infection. It has been described that miRNAs encoded by EBV can downregulate NDRG1, a suppressor of metastasis, to promote EBV-mediated epithelial carcinogenesis, suggesting that NDRG1 plays a negative role in EBV-induced cancer [52]. Another study also showed that NDRG1 restricts hepatitis C virus propagation by regulating lipid droplet formation and viral assembly [53]. On the other hand, a study published this year reported that NDRG1 facilitates influenza A virus replication by suppressing canonical NF-kappa B signaling [54]. All of these studies suggest that NDRG1 may participate in the viral life cycle, but the functions of this protein appear to be diverse. Regardless of the role of NDRG1 in other viruses, the role of this protein in KSHV infection has not been studied. NDRG1 is a member of the NDRG family, currently consisting of NDRG1, NDRG2, NDRG3 and NDRG4. In our study, the omics data of MM and KMM cells showed NDRG1 but not NDRG2-4 is distinctly upregulated in KMM cells, hence we focused on exploring the role of NDRG1 in KSHV infection. We demonstrated that the high expression of NDRG1 in KSHV-positive cells is induced by KSHV infection.

The expression of NDRG1 is regulated by a diverse range of effectors. Many of the effectors are the transcription factors, like EGR-1, Sp-1, Ap-1, and ETS, which are known to regulate NDRG1 expression [33]. The latent phase of KSHV is characterized by the expression of a limited number of viral genes. LANA, a transcription factor, is the most abundant viral protein expressed during latency and is required for various vital functions of KSHV infection [45]. Our data suggested that LANA is essential for upregulation of NDRG1 during KSHV infection. We analyzed the promoter of NDRG1 and found a DNA sequence (CGCTCAGGGCGTGGCGC, -410 of the promoter) similar to LBS1 (CGCCCGGGCATGGGGC), which is the LANA DNA binding motif in TR region of KSHV genome. So it is possible that LANA may regulate NDRG1 expression through interacting with its promoter. We have preliminary data (S9 Fig) indicating that LANA alone can upregulate the mRNA level of NDRG1 slightly but in the meantime the protein level of NDRG1 remains unchanged. This suggests that LANA may regulate the expression of NDRG1 at both transcriptional and post transcriptional levels. Some other viral factors may also be involved in the regulation of NDRG1. The detailed mechanisms for the upregulation of NDRG1 during KSHV infection would be interesting to be further studied in the future.

DNA replication of the KSHV episome along with each cell division event during latency is one of the key components of episome persistence. Perturbation of viral episomal DNA replication will negatively impact viral DNA maintenance [10,11]. LANA, one of the few viral proteins expressed during latency, is essential for genome maintenance as well as viral episomal DNA replication [5,6,8,9,12]. LANA directly binds to the TR region, which is the KSHV latent replication origin region, via its C-terminal end to mediate viral DNA replication [5,8,14–19]. Because LANA lacks the enzymatic activity required for DNA replication, this protein interacts with and recruits diverse cellular proteins for latent viral genome replication, including the origin recognition complex (ORC1-6), poly (ADP-ribose) polymerase 1 (PARP1), minichromosome maintenance complex (MCM), Bromodomain containing 2 (BRD2), H4-specific histone acetylase (HBO1), replication factor C (RFC), topoisomerase II beta (TopoII beta), and structure-specific recognition protein 1 (SSRP1) [5,6,19,23,25–28,55–63]. Despite this LANA-mediated viral DNA replication, some evidence also suggests that latent KSHV DNA replication can be initiated at origins that are not required for LANA replication [12,64]. However, these studies also showed that LANA-mediated DNA replication is the predominant viral DNA replication pattern during latency [64]. Similar to host DNA replication, the main steps in the process of latent KSHV DNA replication include initiation, elongation, and termination. During the elongation step, new viral DNA strands are formed. Recent studies have shown that LANA enhances the loading of PCNA onto the KSHV TR DNA region and enhances the efficiency of latent viral DNA replication. PCNA is a cellular protein that is a component of the DNA replication machinery. This protein encircles the viral DNA and cooperates with host DNA polymerase to increase the efficiency of viral DNA elongation. However, LANA cannot directly interact with PCNA, and LANA recruits PCNA via other proteins, such as RFC and the cellular mitotic checkpoint kinase Bub1. Knockdown of the expression of RFC or Bub1 will reduce the replication and persistence of the viral DNA [23,27]. In our study, we proved that silencing of NDRG1 results in greatly decreased KSHV episome persistence. We further demonstrated for the first time that NDRG1 serves as a scaffold protein, bridging the interaction between LANA and PCNA. NDRG1 mediates the recruitment by LANA of PCNA for loading onto the KSHV genome and enhances LANA-mediated replication, thus affecting the persistence of the KSHV genome. This finding again indicates that the recruitment of PCNA onto the KSHV genome by LANA is important for latent viral DNA replication and persistence.

LANA is 1162-amino-acid protein that can be divided into an N-terminal domain, an internal repeat domain, and a C-terminal domain. The LANA-N region (1–32) interacts with the cellular histones H2A and H2B and is responsible for tethering viral episomes to the host genome [5,65]. Studies have also shown that the LANA-N domain is crucial for LANA-mediated DNA replication and viral genome persistence. The LANA-N region (1–32) recruits host TopoII beta to the TR region was found to be crucial for KSHV DNA replication in KSHV infected cells as well as in transient replication system [25] The LANA-N domain (262–320) interacts with RFC to mediate efficient latent viral replication and persistence [23]. In our study, we found that the LANA-N domain (1–340) interacts with NDRG1 to mediate latent viral replication. This finding is consistent with the finding that the LANA-N domain participates in LANA-mediated DNA replication and viral genome persistence. In addition, to the best of our knowledge, the role of NDRG1 in the nuclei remains unclear. The finding that NDRG1 directly interacts with the nuclear localization protein PCNA, a cellular DNA replication-related protein, indicates a novel function of NDRG1 related to DNA replication in host cells.

In conclusion, our work showed that KSHV infection upregulated the expression of the host cellular protein NDRG1, and silencing of NDRG1 resulted in greatly decreased viral episome persistence. NDRG1 serves as an adaptor that mediates the recruitment by LANA of PCNA for loading onto the KSHV genome and hence affects KSHV genome persistence. Because NDRG1 is critical for efficient LANA-mediated DNA replication and KSHV episome persistence, this work implicates NDRG1 as an attractive target for disruption. In addition, NDRG1 is nondetectable in normal B cells and endothelial cells, which are permissive to KSHV infection. Therefore, strategies that inhibit NDRG1 in KSHV-infected cells may be effective for virus eradication.

## Materials and methods

### Ethics statement

The clinical tissue specimens from patients with KS were collected from Xinjiang Province, northwestern China. The protocols were reviewed and ethically approved by the Institutional Ethics Committee of the First Teaching Hospital of Xinjiang Medical University (Urumqi, Xinjiang, China; Study protocol no. 20082012). Written informed consent was obtained from all participants, and all samples were anonymized. All participants were adults.

### Cell lines and plasmids

The MM, KMM, SLK, iSLK.RGB, iSLK.LANAstop, and HEK293T cell lines were cultured in DMEM (HyClone) supplemented with 10% FBS (HyClone), antibiotics (penicillin and streptomycin, HyClone), and the appropriate selective pressures (hygromycin, 0.5 mg/ml; puromycin, 1.5 μg/ml; G418, 0.5 mg/ml). KSHV-positive B lymphoma cell lines (BCBL1, JSC1, BC3) and KSHV-negative B lymphoma cell lines (DG75, Raji, Loukes, Ramous) were cultured in RPMI 1640 (HyClone) supplemented with 10% FBS (HyClone) and 1% antibiotics (penicillin and streptomycin, HyClone). HUVECs were maintained in EGM (Lonza). All the cell lines were grown at 37°C in a humidified environment supplemented with 5% CO2. We are grateful to Dr. Shoujiang Gao (University of Southern California) for the MM and KMM cell lines [32] and iSLK.LANAstop cell line [46], which produces LANA-depleted KSHV after induction; Dr. Jae Jung (University of Southern California) and Dr. Fanxiu Zhu (The Florida State University) for the SLK and iSLK.RGB cell lines [66]; Dr. Erle S Robertson (University of Pennsylvania, USA) for the BCBL1, JSC1, BC3, DG75, Raji, Loukes, BJAB, and Ramous cell lines. The HEK293T cell line was from our laboratory stock.

The shLANA and control plasmids have been previously reported [67]. Two target-specific shRNAs complementary to NDRG1 (5’-ACC TGC ACC TGT TCA TCA A-3’ and 5’-CGC TGA GGC CTT CAA GTA C-3’) were cloned into the pLKO.1 vector as previously reported. A pLKO.1 vector with a scrambled sequence against NDRG1 was used as a control (5’-GGA ATC TCA TTC GAT GCA TAC-3') [68,69]. Full-length fragments of NDRG1 (NM_001135242.1) and PCNA (NM_002592.2) were amplified from a HEK293T cDNA library. A DNA construct expressing SF-tagged NDRG1 was generated by cloning full-length NDRG1 to a modified pCDH-SF-IRES-Blast vector, which introduced a fragment encoding a tandem Strep-tag II and FLAG peptide. pcDNA3.1-NDRG1 and pcDNA3.1-PCNA were constructed by cloning full-length NDRG1 and PCNA into pcDNA3.1(+), respectively. pcDNA3.1-HA-NDRG1 was constructed by subcloning the HA-NDRG1 fragment into pcDNA3.1(+) from pCMV-HA-NDRG1, which was constructed by subcloning the NDRG1 fragment from pcDNA3.1-NDRG1 into the pCMV-HA vector. Similarly, pcDNA3.1-HA-PCNA was constructed by subcloning the HA-PCNA fragment into pcDNA3.1(+) from pCMV-HA-PCNA, which was constructed by subcloning the NDRG1 fragment from pcDNA3.1-PCNA into the pCMV-HA vector. The plasmids pCAGGS-HA-LANA and pCDH-SF-LANA, encoding FLAG-tagged LANA, were described previously [70,71]. The truncated LANA constructs GST-LANA-N (1–340 aa) and GST-LANA-C (1022–1162 aa) were described previously [72]. GST-fused full-length NDRG1 was obtained by cloning the corresponding fragments into the pGEX-4T-1 vector. The p8TR-gB plasmid, which contains eight TR copies was described previously [50]. All the primers used for gene amplification and qPCR are listed in S5 Table.

### Antibodies and reagents

The following primary antibodies were used: anti-NDRG1 rabbit monoclonal antibody (Abcam, ab124689), anti-PCNA(P10) mouse monoclonal antibody (CST, #2586), anti-PCNA rabbit polyclonal antibody (ABclonal, A4006), anti-α-tubulin antibody (Sigma, T6199), anti-FLAG antibody (Sigma, F1804), anti-CTCF mouse monoclonal antibody (Santa Cruz, sc-271514), anti-CTCF rabbit polyclonal antibody (ABclonal, A1133), anti-LANA rat monoclonal antibody (Advanced Biotechnology Inc, 13-210-1000), and anti-LANA mouse monoclonal antibody (1B5), which was prepared in our laboratory [73]. The secondary antibodies used for western blotting and the IF assay were goat anti-mouse IRDye 800CW (Li-Cor, 926–32210); goat anti-rabbit IRDye 680RD (Li-Cor, 926–68071); goat anti-rabbit antibodies conjugated with Alexa Fluor 488 (Thermo Fisher Scientific, A-11094), 555 (Thermo Fisher Scientific, A27017), and 680 (Thermo Fisher Scientific, A27020); goat anti-mouse antibodies conjugated with Alexa Fluor 488 (Thermo Fisher Scientific, A-11001) and 555 (Thermo Fisher Scientific, A-21422); and goat anti-rat antibodies conjugated with Alexa Fluor 488 (Thermo Fisher Scientific, A-11006). The other reagents used (and their sources) were as follows: anti-FLAG M2 affinity gel (Sigma, A2220), Strep-Tactin Sepharose (IBA, 2-1201-010), desthiobiotin (IBA, 2-1000-001), recombinant protein A agarose (Invitrogen, 15948–014), recombinant protein G agarose (Invitrogen, 15920–010), glutathione Sepharose 4B (GE Healthcare, 17-0756-01), the TNT T7 Quick Coupled transcription/translation system (Promega L1170), Dynabeads M-280 streptavidin (Invitrogen, 11205D).

### Integrative transcriptome and proteome analysis

#### Transcriptome analysis

Approximately 2 million MM or KMM cells were used for transcriptome analysis. RNA was isolated using RNeasy Micro kit (QIAGEN), and RNA integrity was determined by Agilent 2100 Bioanalyzer (Agilent). RNA samples were sent to Shanghai Biotechnology Corporation for Illumina library preparation and RNA-seq (HiSeq 2000, paired-end 2x100bp). About 45 million reads were obtained for each sample. Fastq sequence files were aligned to Rat genome (UCSC rn5) using TopHat v.2.0.6. Differential expression testing, assignment of FPKM and fold change values to genes was performed with CuffDiff v.2.1.1. Differentially expressed genes were selected by FC> = 2 and FDR < = 0.05. MM and KMM microarray data were provided by Dr. SJ. Gao. There are only two replicates for each group. Differentially expressed genes were selected by FC> = 2.

#### Proteome analysis

MM or KMM cells were lysed with RIPA buffer, and the frozen cell lysates were sent to Applied Protein Technology of Shanghai (aptbiotech) for proteome analysis using iTRAQ method [74]. There are three replicates for each group. Briefly, the MM replicates were labeled with iTRAQ tags 113, 114, and 115, and the KMM replicates were labeled with tags 116, 117, and 118. Data acquisition was performed with Q Exactive Hybrid Quadrupole-Orbitrap Mass Spectrometer (Thermo Finnigan). Raw data files were processed in Proteome Discoverer 1.4 (Thermo), and protein identification was performed using the Mascot search engine (version 2.2; Matrix Science) against the *Rat* database (uniprot_Rat.fasta). For each confident identification, the protein included at least one unique peptide. Differentially expressed proteins were selected by p<0.05.

#### Integrative analysis

Overlapping differential genes/proteins among RNA-seq, microarray and iTRAQ were calculated and presented as venn diagram. The list of overlapping differential genes/proteins were provided in S1–S3 Tables.

### Quantitative real-time PCR

To determine the RNA levels or genomic DNA levels, quantitative real-time PCR was used. To analyze RNA levels, the cDNA was reverse transcribed with the Genomic DNA Eraser RT Kit (TaKaRa) from the total RNA extracted from cells harvested with TRIzol reagent (Life Technologies). To analyze DNA levels, total DNA was extracted from the cells with the Genomic DNA Extraction Kit (Tiangen). Relative KSHV episomal copy numbers were calculated by qPCR amplification of the terminal repeats as previously described [61]. qPCR was performed with a SYBR Green Master Mix Kit (Toyobo) on a 7900HT system (Life Technologies). Relative mRNA levels and relative DNA levels were normalized to actin or GAPDH and calculated by the ΔΔCT method. The samples were tested in triplicate. The primers are listed in S5 Table.

### Immunohistochemistry (IHC) and immunofluorescence (IF)

The expression of LANA and NDRG1 in the tissue samples was analyzed by both immunohistochemistry (IHC) and IF as previously described [75]. Primary antibodies consisting of either anti-LANA antibody (1:500) or anti-NDRG1 antibody (1:200) were used. For immunofluorescence, cells were fixed with 4% paraformaldehyde (PFA) for 30 min and permeabilized with 0.2% Triton X-100 for 30 min. Next, the cells were blocked in 10% goat serum (Life Technologies) for 1 hr, followed by incubation with anti-NDRG1 (1:100), anti-PCNA (1:100) or anti-LANA (1:250) antibody overnight at 4°C and further staining with the secondary antibodies at a 1:1,000 dilution for 1 hr. Cell nuclei were stained with DAPI (Sigma, D5942). Slides were photographed using a digital camera and software (FV-1200; Olympus).

### KSHV preparation and infection

Wild-type KSHV virions were acquired by inducing iSLK.BAC16.RGB cells with doxycycline (DOX) and valproic acid as described previously [76]. Briefly, iSLK.BAC16.RGB cells were induced with DOX (1 g/ml) and valproic acid (1 mM) in the absence of hygromycin, puromycin, and G418. Four days later, the supernatant was collected by centrifugation (1,500×*g* at 4°C for 30 min), followed by filtration. Virus particles were precipitated with 8.8% polyethylene glycol 6000 by centrifugation (1,500×*g* at 4°C for 1 hr). The MOI of the concentrated viral stock was determined by infection of HEK293T cells for 24 hr. Infection of SLK cells or HUVECs was achieved by centrifugation at 1,250×*g* at 37°C for 2 hr after the addition of concentrated virus to the medium, and the medium was changed at 2 hpi. UV-inactivated KSHV (UV-KSHV) was used in this study. Replication-incompetent KSHV was prepared by exposing wild-type KSHV for 30 min under UV light as described earlier [77]. Wild-type and LANA-depleted KSHV were also used in this study. LANA-depleted KSHV virions were acquired from iSLK.LANAstop cells, which produce LANA-depleted KSHV after induction. Titration of KSHV stocks was performed as described previously [46].

### Transfection and transduction

To transduce SLK, KMM, and BCBL1 cells, pCDH plasmids were packaged in HEK293T cells by cotransfection with the Δ8.9 packaging plasmid and a plasmid expressing vesicular stomatitis virus G protein (pVSV-G) as described previously [76]. Three days later, the supernatant was collected and cleared by filtration. Lentiviral particles were precipitated with 7% polyethylene glycol 6000 and pelleted by centrifugation at 1,500×*g* for 1 hr at 4°C. The transduction of SLK cells was achieved by centrifugation at 1,250×*g* for 2 hr after addition of the concentrated virus to the medium. The transduction of KMM and BCBL1 cells was achieved by addition of the concentrated virus to the medium for 24 hr. HEK293T cells were transfected with polyethylenimine as described previously [67]. BJAB cells were transfected by nucleofection. Cells were washed with PBS and resuspended in 100 μl of transfection buffer from the Ingenio Kit (MIR 50118, Mirus) and nucleofected with plasmids using the Amaxa Nucleofector II system (Lonza) according to the manufacturer’s instructions.

### Fluorescence *in situ* hybridization analysis (FISH)

DNA FISH assays were performed as previously described [8,63], with minor modifications. Briefly, cells were fixed in 4% PFA at room temperature for 30 min and then fixed in 70% ethanol overnight at -20°C. Fixed cells were permeabilized with 0.5% Triton X-100. Subsequently, the cells were treated with 100 μg/ml of RNase A in 2×SSC (1×SSC contains 0.15 M NaCl plus 0.015 M sodium citrate). The slides were overlaid with *in situ* hybridization solution containing 20 ng of KSHV TR probe labeled with DIG using the DIG High Prime DNA labeling system (Roche) according to the manufacturer’s instructions. After denaturation of the DNA at 93°C for 5 min, the slides were incubated for 24 hr at 42°C, washed in 2×SSC for 30 min at 45°C, and blocked in TNB (0.1 M Tris-HCl (pH 7.5), 0.15 M NaCl, 0.5% blocking reagent) at room temperature for 30 min. The slides were then incubated with anti-DIG antibody (2 μg/ml) for 2 hr and with goat-anti-mouse 555 (1:1000) for 1 hr at room temperature. The slides were washed and counterstained with DAPI, followed by mounting with antifade and visualizing with a digital camera and software (FV-1200; Olympus).

### Tandem affinity purification-mass spectrometry (TAP-MS)

TAP-MS was used to identify NDRG1 protein complexes. The procedures were as described previously [73,78]. Briefly, SF-tagged NDRG1 or SF-tagged vector was overexpressed in iSLK.RGB cells. Cells were lysed, and the extract was loaded into a Strep-Tactin Sepharose column (IBA). The column was washed with buffer W (50 mM Tris (pH 7.9), 100 mM KCl, 10% glycerol, 0.2 mM EDTA, 0.5 mM DTT, 0.1% Triton-X100, 0.2 mM PMSF) and eluted with buffer E (buffer W containing 2.5 mM D-desthiobiotin). The elute was then subjected to a second round of affinity purification on an anti-FLAG M2 affinity gel for 4 hr at 4°C. The beads were washed with buffer W 4 times and eluted with 3×FLAG peptide in buffer W. The elute was monitored by SDS-PAGE and subjected to mass spectrometry.

### Coimmunoprecipitation (co-IP) and immunoblotting

Cells were lysed in radio immunoprecipitation assay (RIPA) buffer (50 mM Tris-HCl (pH 7.4), 150 mM NaCl, 0.5% Triton X-100, 1 mM PMSF) for 1 hr on ice with brief vortexing every 15 min. The cells were then ere centrifuged at 12,000×*g* at 4°C for 30 min to remove cell debris. Five percent of the cell lysates were kept as inputs. The remainder was precleared with protein A- or protein G-coupled Sepharose (Life Technologies) for 2 hr at 4°C and then immunoprecipitated with the corresponding antibodies overnight at 4°C. Immunoprecipitates were washed four times with RIPA buffer and then boiled in SDS loading buffer for western blot analysis. For western blotting, protein samples were analyzed by SDS-PAGE and transferred onto nitrocellulose membranes, followed by blocking and probing with the indicated antibodies for detection.

### Protein purification and *in vitro* binding assay (GST pull down)

The procedures were performed as described previously [70]. Briefly, GST or GST fusion proteins were expressed in *Escherichia coli* strain BL21 (DE3), which was grown in LB medium at 37°C to exponential phase and cultured overnight at 16°C after induction with isopropyl thiogalactopyranoside (IPTG). The cells were harvested and resuspended in ice-cold PBS, followed by sonication lysis (Sonics; 3/5 s, pulse cycle; 35%, amplitude). The cell lysates were centrifuged at 12,000×*g* to obtain the supernatant, which was combined with Sepharose 4B-glutathione resin for affinity purification according to the manufacturer’s instructions. *In vitro* translated proteins produced by the TNT-coupled transcription/translation system were incubated with purified GST-fusion-protein-bound beads in RIPA buffer for 12 hr. After washing with RIPA buffer four times, the pull-down products were analyzed by western blotting.

### *In vitro* DNA pull-down assay

To determine whether NDRG1 mediates PCNA loading onto TR DNA in the presence of LANA, a TR biotin-labeled DNA pull-down assay was adapted from previously described methods [23,79]. Briefly, the biotin-labeled 801-bp TR DNA fragment was amplified by PCR using 5’-biotin-labeled primers and the p8TR plasmid as the template. The forward primer sequence was 5’ GCGCCTGGTCCCGCCCCCGCCCGC 3’, and the reverse primer sequence was 5’ CGGCCGCGCCGGGCCCTGAGGCGGC 3’. Ten million cells were lysed in RIPA buffer (50 mM Tris-HCl (pH 7.4), 150 mM NaCl, 0.5% Triton X-100, 1 mM PMSF) for 1 hr on ice, vortexing very briefly every 15 min. Cells were centrifuged at 12,000×*g* at 4°C for 30 min to remove cell debris. Five percent of the cell lysate was kept as input. The remainder of the cell lysate was incubated with the purified biotin-TR DNA fragment (100 ng) supplemented with 1 μg/ml poly dI/dC for 4–5 hr at 4°C and then immobilized on 15 μg of streptavidin beads following the manufacturer’s protocol (Dynabeads M-280 streptavidin; Invitrogen) for 4–5 hr at 4°C. Following this immobilization, the supernatant was removed. The beads were washed four times with PBS and then boiled in SDS loading buffer for western blot analysis.

### Chromatin immunoprecipitation (ChIP)

ChIP assays were performed as described previously [23,70]. Briefly, cells were fixed in medium with 1% formaldehyde for 30 min (LANA, NDRG1 and PCNA) at room temperature and quenched with 0.125 M glycine. For revealing differences in PCNA loading on TR region with or without NDRG1, cells were especially immersed in hypotonic buffer containing 0.1% Triton X-100 for 10 min before fixation [80]. After fixation, the cells were washed with PBS twice and lysed in SDS lysis buffer (50 mM HEPES, 1 mM EDTA, 1% SDS, 1 mM PMSF) supplemented with protease inhibitor cocktail for 30 min on ice. The lysates were subjected to sonication to obtain 200-500-bp DNA fragments (Sonics; 2/6 s, pulse cycle; 30–35%, amplitude) and then centrifuged to obtain the supernatants. The supernatants were diluted with RIPA buffer. Samples were precleared with pretreated protein A or G beads (1 mg/ml BSA, 1 mg/ml sperm DNA, 20% beads) for 2 hr at 4°C. A small fraction of the supernatants was kept as input, and the remainder was divided into groups according to the experiment. The aliquots were incubated with pretreated protein A or G beads and the corresponding antibody overnight at 4°C. After extensive washing with RIPA buffer, wash buffer (20 mM Tris-HCl (pH 8.0), 1 mM EDTA, 250 mM LiCl, 0.5% NP-40, 1 mM PMSF) and TE buffer (10 mM Tris-HCl (pH 8.0), 1 mM EDTA), three to four times each, the beads were resuspended in TE buffer. The resuspended beads were subjected to RNaseA and proteinase K digestion, and the crosslinking was reversed at 65°C overnight. The DNA was recycled using a DNA purification kit.

### Transient DNA replication assay

Transient replication assays in uninfected cells were used to assess LANA-mediated DNA replication, and the procedures were performed as described previously [50]. In brief, for the DNA replication assay performed in BJAB cell lines, ten million cells were cotransfected by nucleofection with 6 μg of p8TR-gB, 6 μg of NDRG1 and 6 μg of LANA or empty vector plasmids. After twenty-four hours of transfection, five million cells were collected and used to normalize the transfection efficiency, and the other cells were further cultured. Seventy-two hours post transfection, 10 million cells were collected and subjected to DNA extraction by Hirt’s method [81]. For the DNA replication assay in HEK293T-LANA cells stably expressing LANA, cells in 10-cm dishes were cotransfected with 20 μg of p8TR and 10 μg of NDRG1 or an empty vector plasmid. Similarly, after twenty-four hours of transfection, cells were collected and used to normalize the transfection efficiency, and the other cells were further cultured. Seventy-two hours post transfection, the cells were collected and subjected to DNA extraction by Hirt’s method. To detect replicated p8TR-gB, the Hirt DNA was digested overnight at 37°C with *Dpn*I in NEB buffer #2, which was followed by exonuclease III (*Exo*III) treatment for 30 min at 37°C. The digested DNA was assayed for replication using real-time PCR as previously described [50]. The results represent the average values obtained from three experiments.

### Plasmid maintenance assay

For the plasmid maintenance assay performed in BJAB cells, ten million cells were cotransfected by nuclofection with 6 μg of p8TR-gB, 6 μg of NDRG1 and 6 μg of LANA or empty vector plasmids. For the plasmid maintenance assay performed in HEK293T-LANA cells, 20 μg of the p8TR plasmid was transfected into HEK293T-LANA cells along with 10 μg of NDRG1 or vector plasmids. After twenty-four hours of transfection, five million cells were collected and used to normalize the transfection efficiency, and the other cells were further cultured for several days. To detect total p8TR-gB DNA levels, the cells were collected on days 1, 2, 3, and 4, followed by extraction of genomic DNA. Relative TR DNA levels of the p8TR plasmid were calculated by qPCR amplification of the TRs, normalized to GAPDH levels, and analyzed by the ΔΔCT method as previously described [61]. TR plasmids can be maintained long term in subpopulations in the absence of drug selection [51]. To test whether NDRG1 assists LANA retain TR in the long term, part of the transfected HEK293T-LANA cells described above were further cultured without drug selection for 10 days. As described above, the cells were collected and analyzed by qPCR [61].

### Statistical analysis

Data are presented as the mean ± standard deviation (x ± SD). The level of significance was set at *P-*value<0.05, as determined by Student’s *t-*tests. All experiments were carried out independently at least three times, and representative results are presented.

## Supporting information

S1 Fig Differentially expressed candidate genes in KMM and MM cells. Candidate genes from omic analyses are Tagln, Anxa3, Ocm2, Rcn2, Il1rn,Thop1, Cyr61, Klhl41, Pdlim7, Fkbp9, Nagk, Ndrg1, Dysf, Gsdmd, C1qtnf5, and Rcn3. The RNA levels of these genes in KMM and MM cells were measured by qPCR, respectively. qPCR data were normalized to endogenous actin expression and were shown as mean ± SD, n = 3.
(TIF)

S2 Fig The efficiency of infection of HUVEC and SLK with KSHV. SLK cells were infected with KSHV.BAC16.RGB (MOI, 5) and HUVEC cells were infected with KSHV.BAC16.RGB (MOI,10). Fluorescence was visualized by using an inverted fluorescence microscope at 48 hpi. KSHV-infected cells are indicated by red fluorescence.
(TIF)

S3 Fig LANA is essential for maintenance of NDRG1 in PEL cells. JSC1s, KSHV positive PEL cells, were transduced with lentiviruses containing a LANA RNA interference plasmid (shLANA) or a vector plasmid (shcon). The expression of LANA and NDRG1 in cells were detected by western blotting.
(TIF)

S4 Fig The efficiency of infection of SLK-shcon and SLK-shLANA cells with KSHV. SLK-shon and SLK-shLANA cells were infected with KSHV.BAC16.RGB (MOI, 5), and fluorescence was visualized by using an inverted fluorescence microscope at 0, 12, 24, 36, 48 hpi. KSHV-infected cells are indicated by red fluorescence.
(TIF)

S5 Fig The RNA levels of LANA and RTA were decreased in the absence of NDRG1 in KMM cells. Total RNA were collected form KMM-shcon, KMM-shNDRG1-1#, and KMM-shNDRG1-2# cells. The RNA levels of LANA and RTA were determined by qPCR. qPCR data were normalized to the level of endogenous GAPDH in each group. Data were shown as mean ± SD, n = 3, **p<0.01, ***p<0.001.
(TIF)

S6 Fig Silencing NDRG1results in reduced TR DNA in KSHV infected cells. KMM-shcon and KMM-shNDRG1-1# cells were hybridized with DIG-labeled KSHV TR probe. Cells were then incubated with anti-DIG antibody followed by incubating with goat-anti-mouse 555 (red). Cells were also counterstained with DAPI (blue). Scale bars represent 5μm.
(TIF)

S7 Fig Endogenous LANA-specific association of NDRG1 and PCNA in PEL cells. Co-IP of endogenous LANA, NDRG1, and PCNA in BCBL1 cells. Cell lysates were subjected to IP with anti-LANA mouse monoclonal antibody(1B5), or anti-CTCF mouse monoclonal antibody, or mouse IgG controls. Purified proteins along with input samples were detected by western blotting with anti-LANA, anti-CTCF, anti-NDRG1, and anti-PCNA antibodies. In order to exclude the contamination of the anti-LANA IPs with KSHV episomal chromatin, we have added benzonase nuclease in cell lysis before IPs.
(TIF)

S8 Fig The full-length western blot images for the antibodies and molecular weight markers of in vitro TR biotin-labeled DNA pull-down assay. NDRG1 and/or LANA was transfected into BJAB cells. After 24 hr, cells were lysed and five percent of the cell lysates were kept as inputs, and the remainder was incubated with purified biotin-TR DNA fragment and immobilized to streptavidin beads. The inputs and the pulled down products were analyzed by western blotting. The Odyssey^TM^ Western Blotting assays were performed as described in the webpage (www.licor.com). Briefly, cell lysates were resolved by SDS-PAGE and transferred to nitrocellulose membrane. The blot was probed with primary antibodies (mouse anti-LANA antibody, or mouse anti-Tubulin and rabbit anti-NDRG1antibodies, or rabbit anti-PCNA antibody) followed by detection with IRDye 800CW goat anti-mouse IgG and IRDye 680RD goat anti-rabbit IgG. For antibodies labeled with IR 680, select channel 700 (red) and for antibodies labeled with IR 800, select channel 800 (green) via Odyssey infrared imagine system (LI-COR Biosciences) to scan the membranes.
(TIF)

S9 Fig The mRNA and protein levels of NDRG1 in ectopic expression of LANA in SLK cells. The plasmids pCAGGS-HA-LANA and pCAGGS-HA vector were transfected into SLK cells. After 48hr, cells were collected for detecting the RNA and protein levels for NDRG1 via qPCR (A) and western blotting (B). qPCR data were normalized to the level of endogenous GAPDH in each group. Data were shown as mean ± SD, n = 3, *p<0.05.
(TIF)

S1 Table Differentially expressed candidate genes by comparing microarray and iTRAQ database. (XLSX)

S2 Table Differentially expressed candidate genes by comparing RNA-seq and iTRAQ database. (XLSX)

S3 Table Differentially expressed candidate genes by comparing microarray, RNA-seq, and iTRAQ database. (XLSX)

S4 Table NDRG1-interacting nucleoproteins identified in TAP-MS. (XLSX)

S5 Table Primers for PCR amplification and analysis. (DOCX)
