# Supplementary figures and images for: NDRG1 facilitates the replication and persistence of Kaposi’s sarcoma-associated herpesvirus by interacting with the DNA polymerase clamp PCNA
Source: PLoS Pathog. 2019 Feb 27;15(2):e1007628. doi: 10.1371/journal.ppat.1007628 (PMC6411202; doi:10.1371/journal.ppat.1007628)

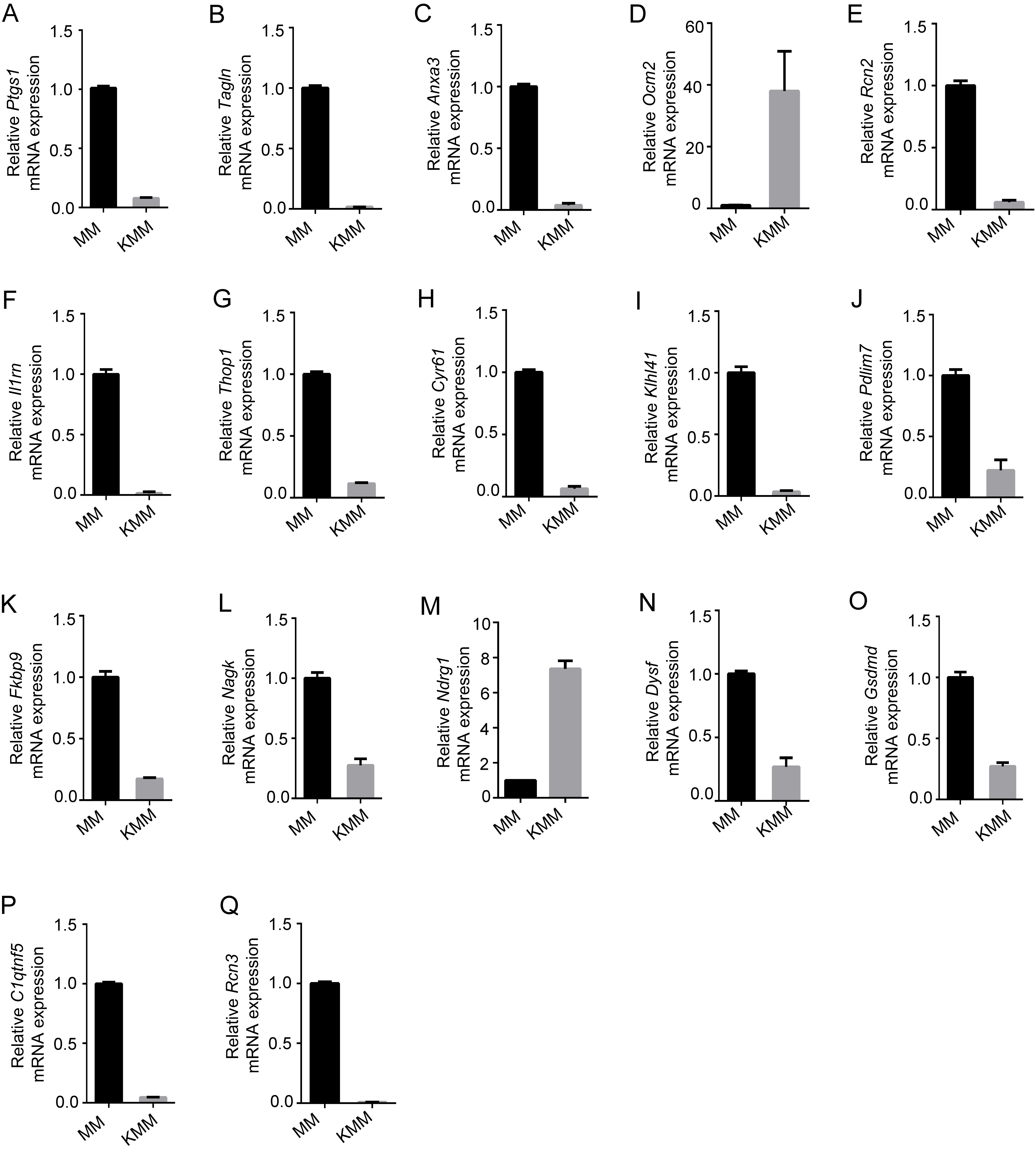

Supplement: S1 Fig — Candidate genes from omic analyses are Tagln, Anxa3, Ocm2, Rcn2, Il1rn,Thop1, Cyr61, Klhl41, Pdlim7, Fkbp9, Nagk, Ndrg1, Dysf, Gsdmd, C1qtnf5, and Rcn3. The RNA levels of these genes in KMM and MM cells were measured by qPCR, respectively. qPCR data were normalized to endogenous actin expression and were shown as mean ± SD, n = 3. (TIF) [file ppat.1007628.s001.tif]

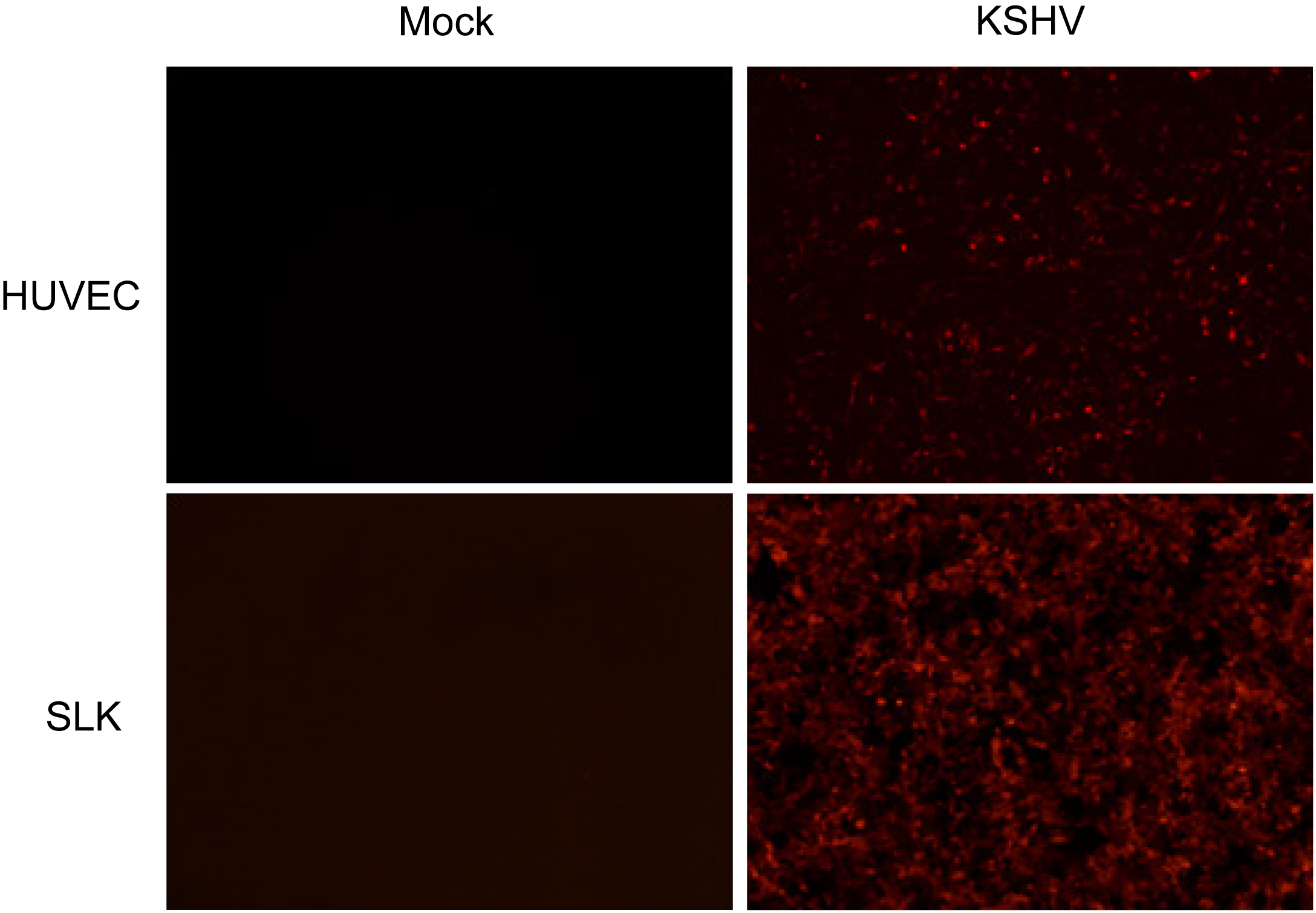

Supplement: S2 Fig — SLK cells were infected with KSHV.BAC16.RGB (MOI, 5) and HUVEC cells were infected with KSHV.BAC16.RGB (MOI,10). Fluorescence was visualized by using an inverted fluorescence microscope at 48 hpi. KSHV-infected cells are indicated by red fluorescence. (TIF) [file ppat.1007628.s002.tif]

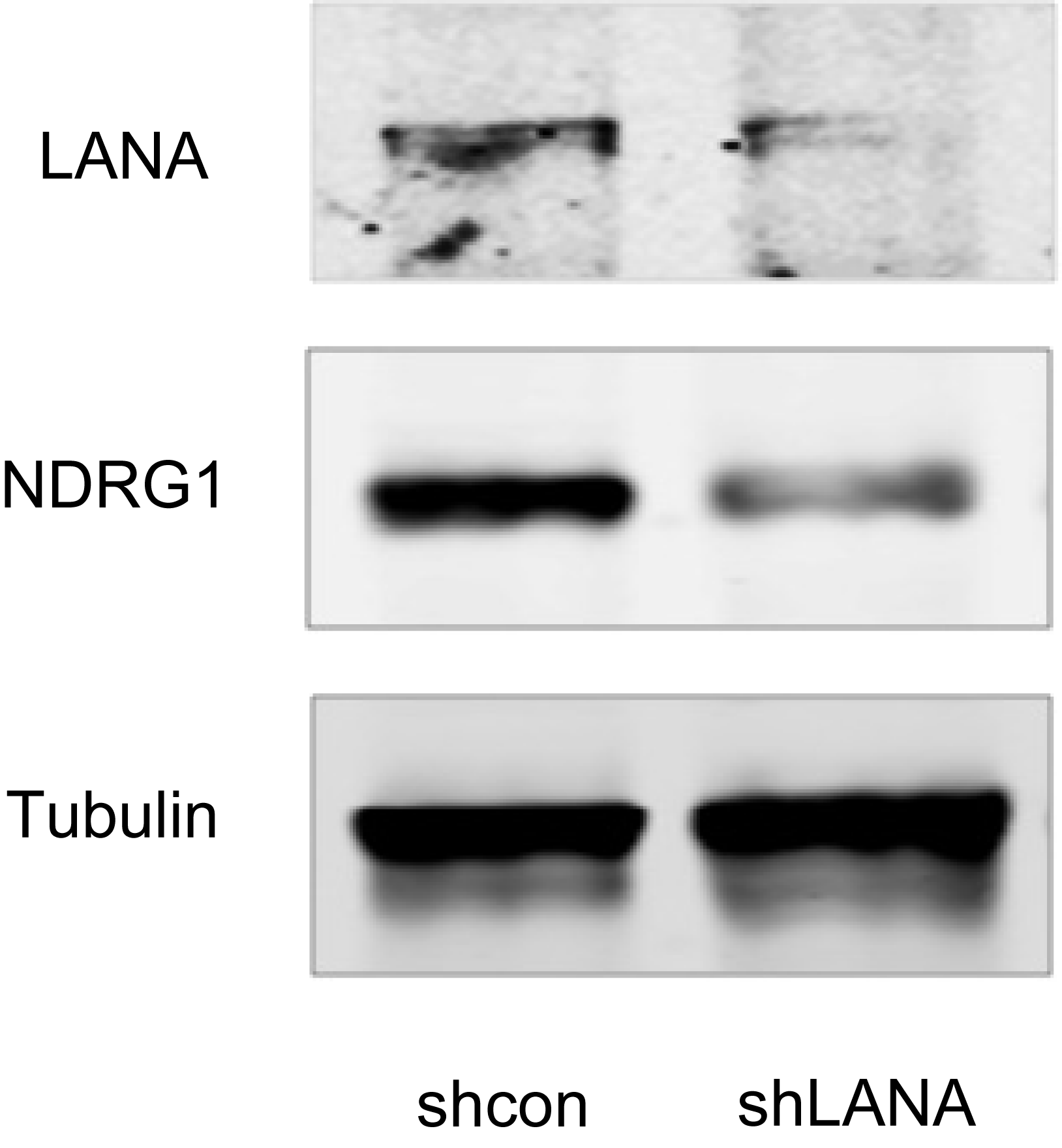

Supplement: S3 Fig — JSC1s, KSHV positive PEL cells, were transduced with lentiviruses containing a LANA RNA interference plasmid (shLANA) or a vector plasmid (shcon). The expression of LANA and NDRG1 in cells were detected by western blotting. (TIF) [file ppat.1007628.s003.tif]

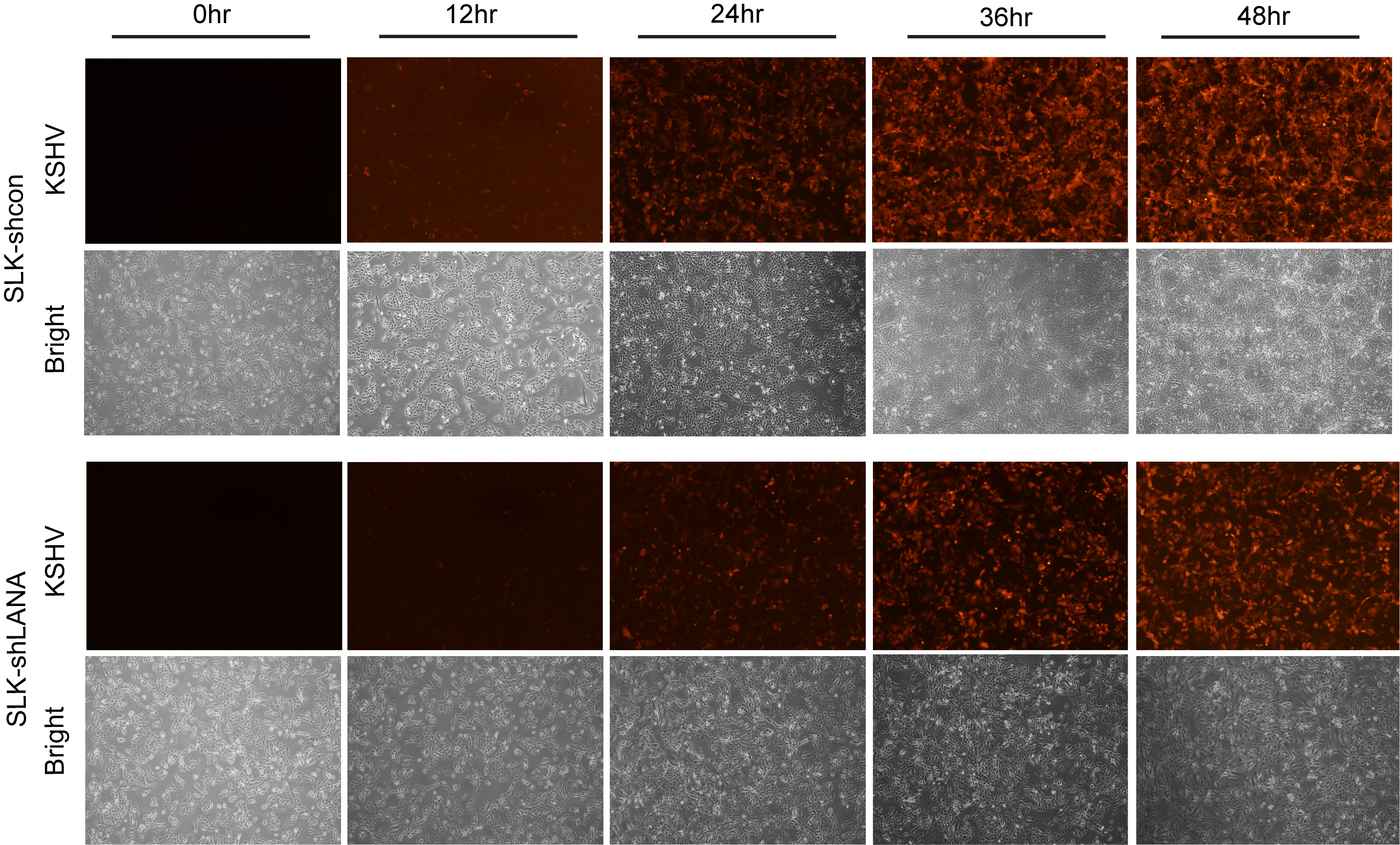

Supplement: S4 Fig — SLK-shon and SLK-shLANA cells were infected with KSHV.BAC16.RGB (MOI, 5), and fluorescence was visualized by using an inverted fluorescence microscope at 0, 12, 24, 36, 48 hpi. KSHV-infected cells are indicated by red fluorescence. (TIF) [file ppat.1007628.s004.tif]

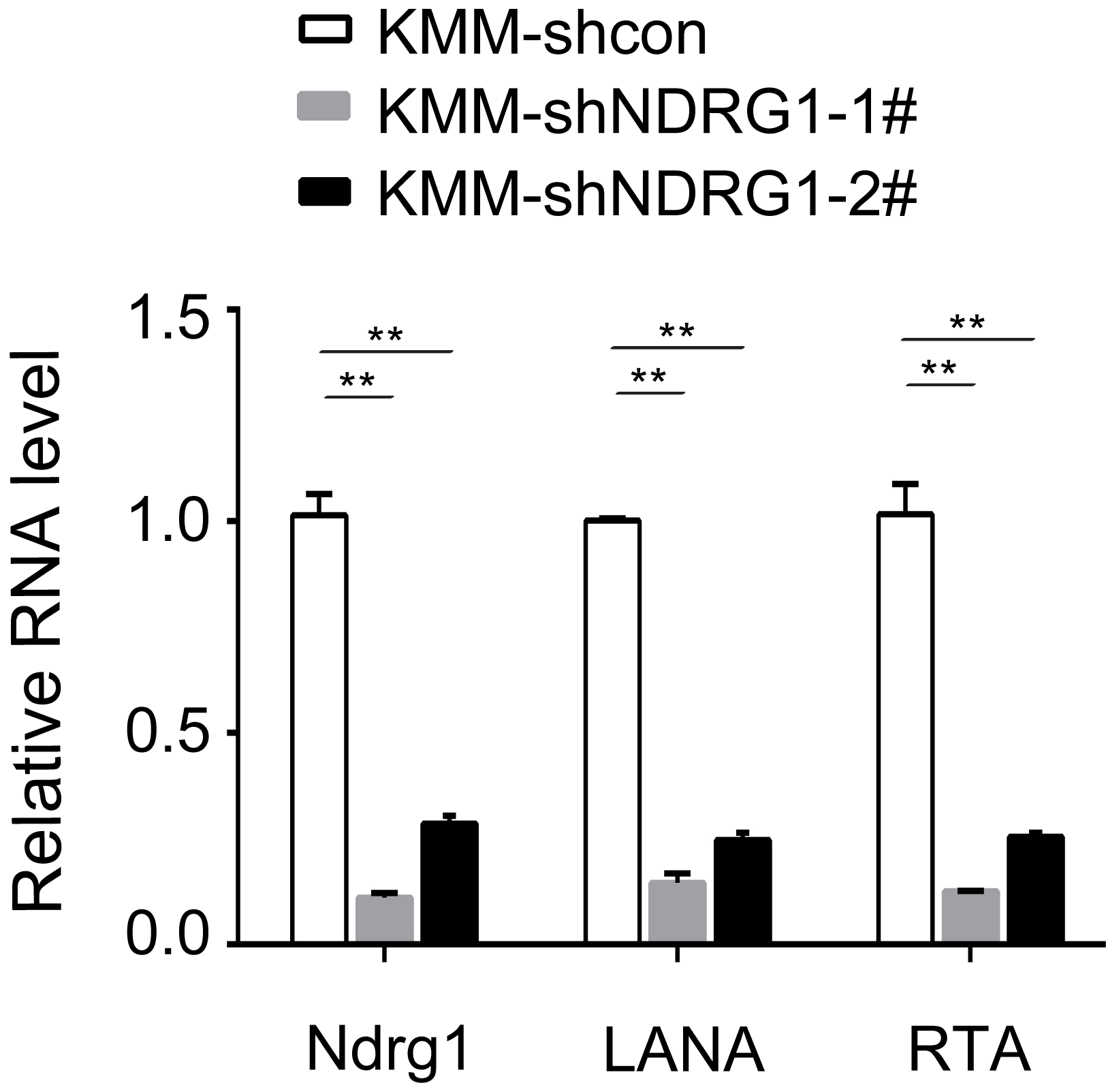

Supplement: S5 Fig — Total RNA were collected form KMM-shcon, KMM-shNDRG1-1#, and KMM-shNDRG1-2# cells. The RNA levels of LANA and RTA were determined by qPCR. qPCR data were normalized to the level of endogenous GAPDH in each group. Data were shown as mean ± SD, n = 3, **p<0.01, ***p<0.001. (TIF) [file ppat.1007628.s005.tif]

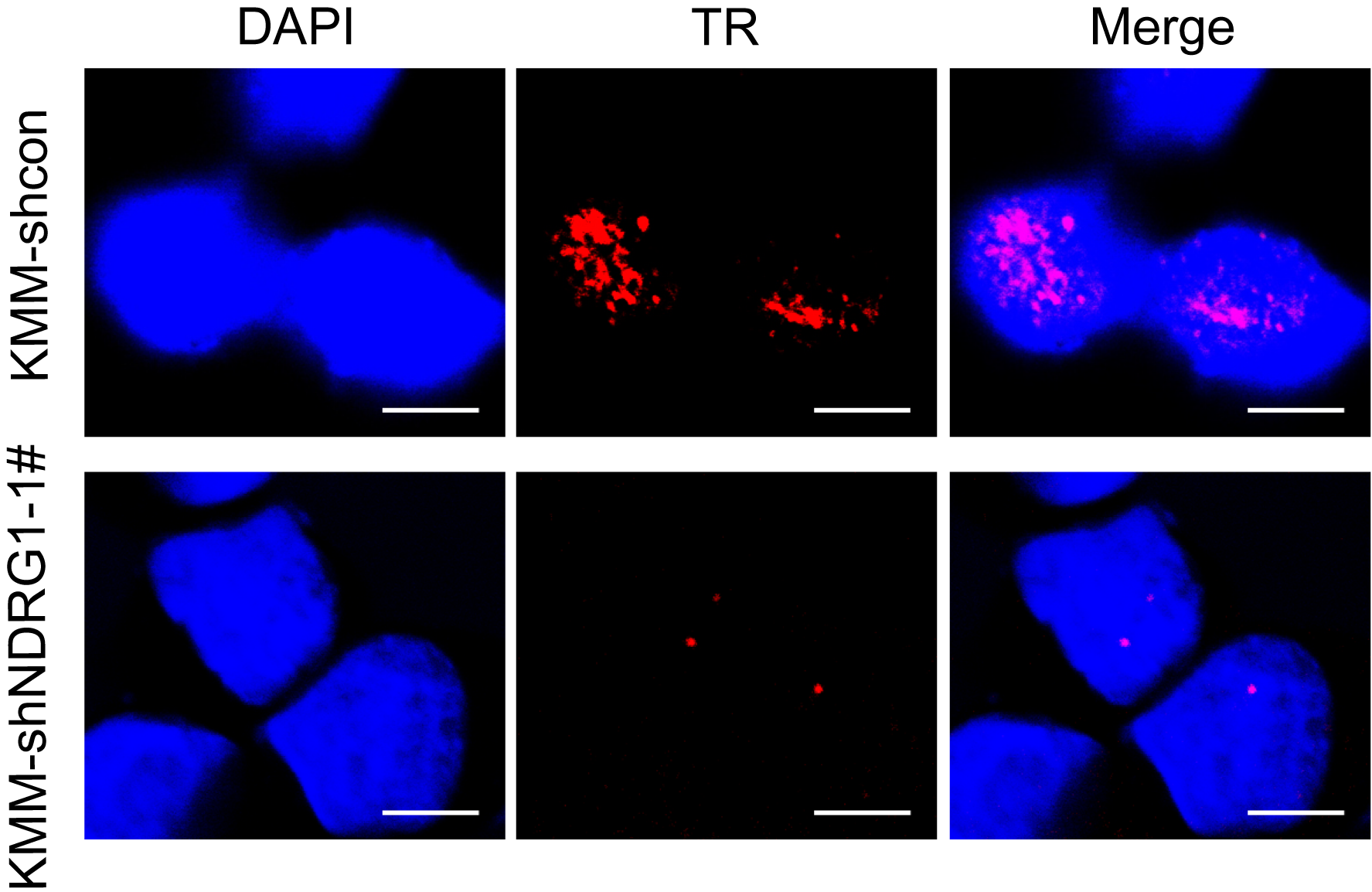

Supplement: S6 Fig — KMM-shcon and KMM-shNDRG1-1# cells were hybridized with DIG-labeled KSHV TR probe. Cells were then incubated with anti-DIG antibody followed by incubating with goat-anti-mouse 555 (red). Cells were also counterstained with DAPI (blue). Scale bars represent 5μm. (TIF) [file ppat.1007628.s006.tif]

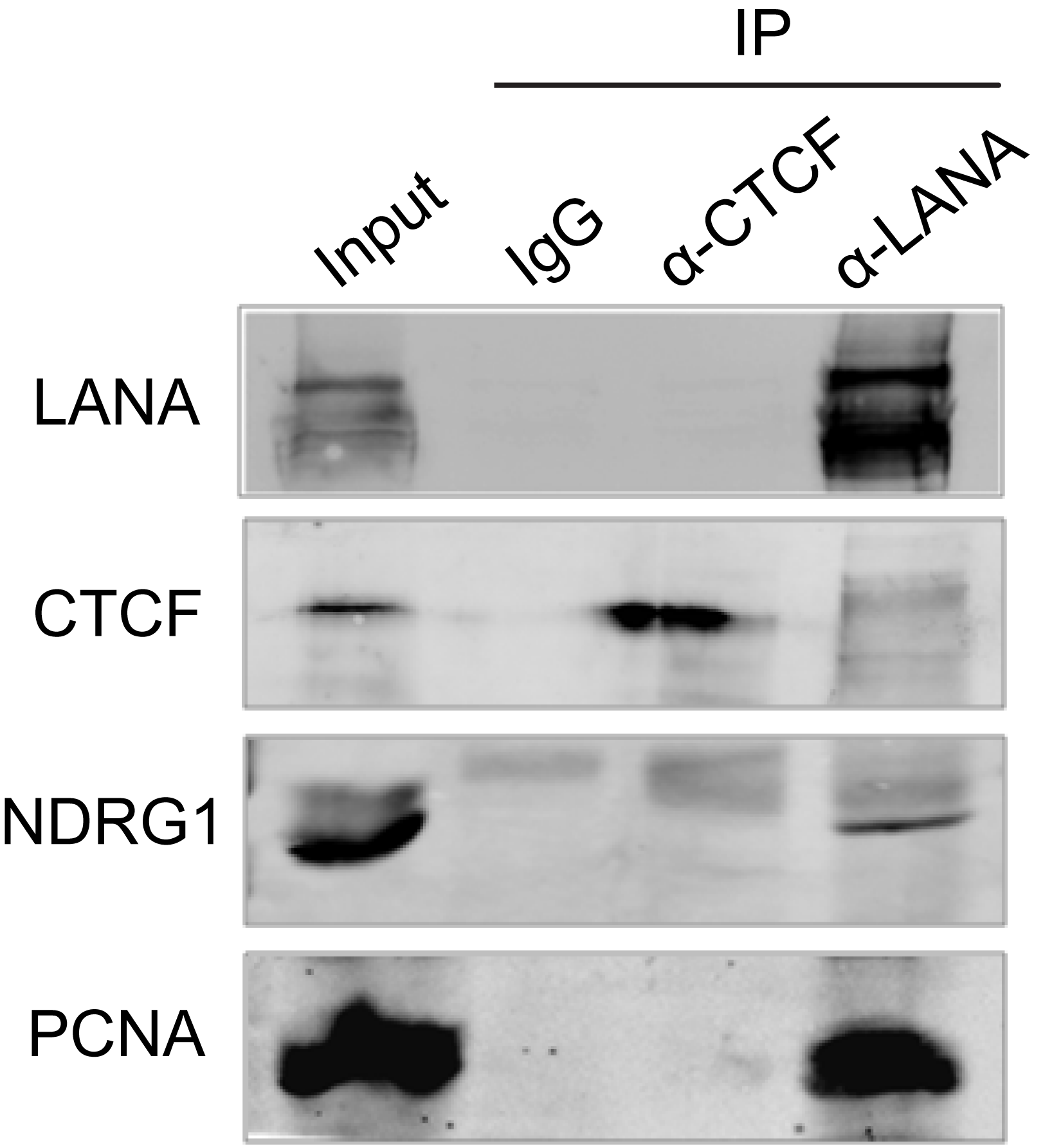

Supplement: S7 Fig — Co-IP of endogenous LANA, NDRG1, and PCNA in BCBL1 cells. Cell lysates were subjected to IP with anti-LANA mouse monoclonal antibody(1B5), or anti-CTCF mouse monoclonal antibody, or mouse IgG controls. Purified proteins along with input samples were detected by western blotting with anti-LANA, anti-CTCF, anti-NDRG1, and anti-PCNA antibodies. In order to exclude the contamination of the anti-LANA IPs with KSHV episomal chromatin, we have added benzonase nuclease in cell lysis before IPs. (TIF) [file ppat.1007628.s007.tif]

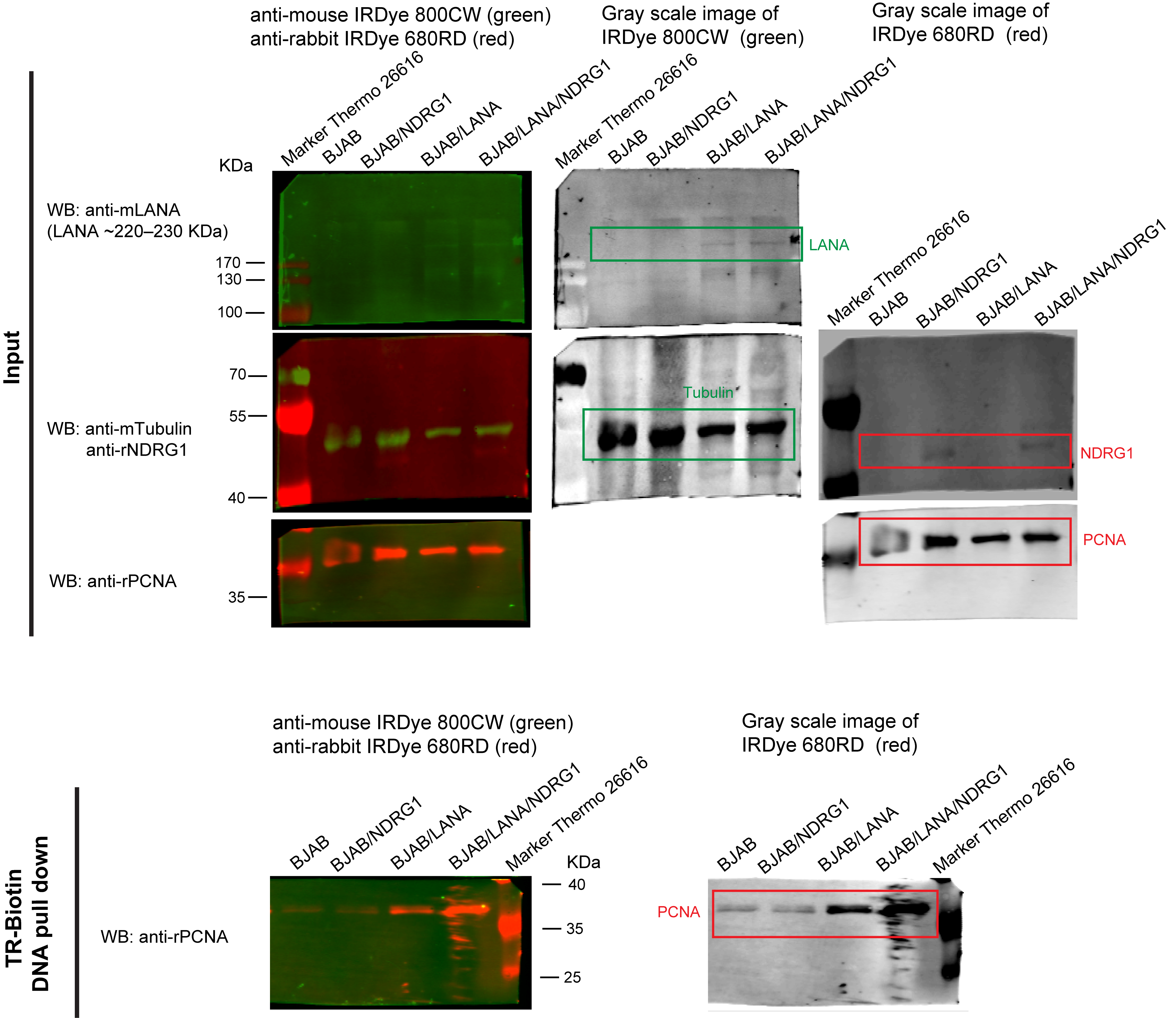

Supplement: S8 Fig — NDRG1 and/or LANA was transfected into BJAB cells. After 24 hr, cells were lysed and five percent of the cell lysates were kept as inputs, and the remainder was incubated with purified biotin-TR DNA fragment and immobilized to streptavidin beads. The inputs and the pulled down products were analyzed by western blotting. The OdysseyTM Western Blotting assays were performed as described in the webpage (www.licor.com). Briefly, cell lysates were resolved by SDS-PAGE and transferred to nitrocellulose membrane. The blot was probed with primary antibodies (mouse anti-LANA antibody, or mouse anti-Tubulin and rabbit anti-NDRG1antibodies, or rabbit anti-PCNA antibody) followed by detection with IRDye 800CW goat anti-mouse IgG and IRDye 680RD goat anti-rabbit IgG. For antibodies labeled with IR 680, select channel 700 (red) and for antibodies labeled with IR 800, select channel 800 (green) via Odyssey infrared imagine system (LI-COR Biosciences) to scan the membranes. (TIF) [file ppat.1007628.s008.tif]

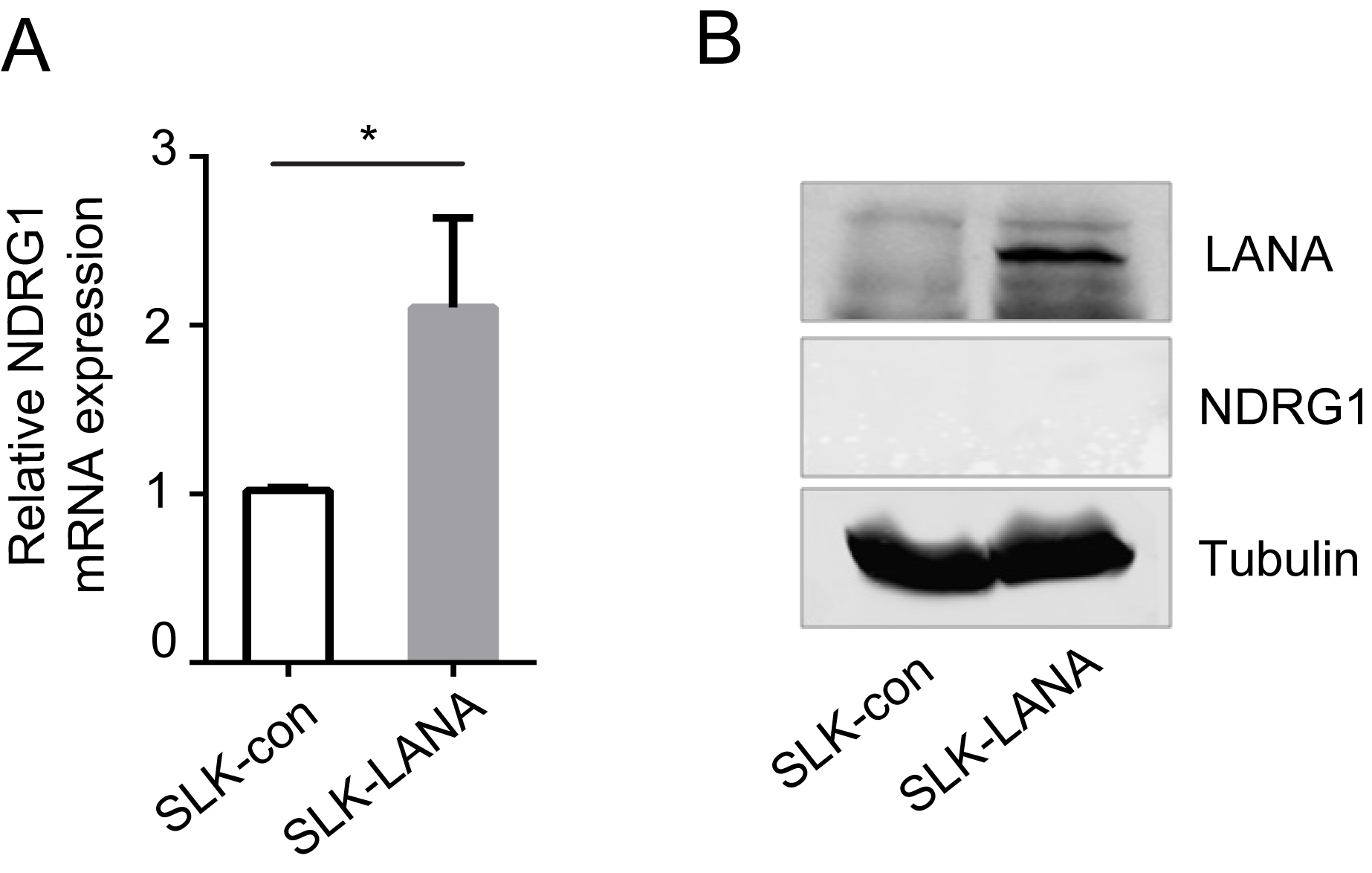

Supplement: S9 Fig — The plasmids pCAGGS-HA-LANA and pCAGGS-HA vector were transfected into SLK cells. After 48hr, cells were collected for detecting the RNA and protein levels for NDRG1 via qPCR (A) and western blotting (B). qPCR data were normalized to the level of endogenous GAPDH in each group. Data were shown as mean ± SD, n = 3, *p<0.05. (TIF) [file ppat.1007628.s009.tif]
