# Supplementary material for: NDRG1 facilitates the replication and persistence of Kaposi’s sarcoma-associated herpesvirus by interacting with the DNA polymerase clamp PCNA
Source: PLoS Pathog. 2019 Feb 27;15(2):e1007628. doi: 10.1371/journal.ppat.1007628 (PMC6411202; doi:10.1371/journal.ppat.1007628)
Supplement: S5 Table — (DOCX) [file ppat.1007628.s014.DOCX]

| Primer Name | Sequence (5’ - 3’) |
| --- | --- |
| SF-NDRG1-F | AGCCTCGAGATGTCTCGGGAGATGCA |
| SF-NDRG1-R | GGCGAATTCGCAGGAGACCTCCATG |
| pCDNA3.1-NDRG1-F | CTCGGATCCATGTCTCGGGAGAT |
| pCDNA3.1-NDRG1-R | GGGCTCGAGCTAGCAGGAGACCTCC |
| pCDNA3.1-PCNA-F | CGCTCGAGATGTTCGAGGCGC |
| pCDNA3.1-PCNA-R | CCTCTAGACTAAGATCCTTCTTCATCCTCGATC |
| pCMV-HA-NDRG1-F | GGCTCGAGAATGTCTCGGGA |
| pCMV-HA-NDRG1-R | CCGGATCCCTAGCAGGAGA |
| pCMV-HA-PCNA-F | GGCTCGAGAATGTTCGAGGCG |
| pCMV-HA-PCNA-R | CCAGATCTCTAAGATCCTTCTTCATCCTCG |
| pCDNA3.1-HA-NDRG1-F | GGCTCGAGCCACCATGTACC |
| pCDNA3.1-HA-NDRG1-R | CCCTCTAGACTAGCAGGAGACCTCC |
| pCDNA3.1-HA-PCNA-F | GGCTCGAGCCACCATGTA |
| pCDNA3.1-HA-PCNA-R | CCCTCTAGACTAAGATCCTTCTTCA |
| GST-NDRG1-F | GGGGATCCATGTCTCGGGAGATGC |
| GST-NDRG1-R | CCCTCGAGGCAGGAGACCTCCAT |
| qGAPDH-F | CTGGGCTACACTGAGCACC |
| qGAPDH-R | AAGTGGTCGTTGAGGGCAATG |
| qACTIN-F | GCACGGCATCGTCACCAACT |
| qACTIN-R | CATCTTCTCGCGGTTGGCCT |
| qRat-Ndrg1-F | CAACATTTTGCGGTCTGCCA |
| qRat-Ndrg1-R | TCCCCATGCCAATGACACTC |
| qRat-Cox1-F | AAGTACTCATGCGCCTGGTACTC |
| qRat-Cox1-R | CATGTGCTGTGTTGTAGGTTGGA |
| qRat-Tagln1-F | AGGTGTGGCTGAAGAATGGCG |
| qRat-Tagln1-R | TCTTCGTGACTCCATAATCCTC |
| qRat-ANXA3-F | CTCTGCCCCAGAGATGTAGC |
| qRat-ANXA3-R | ACTCGTGGGGTGACCATTTC |
| qRat-Ocm2-F | ATGGTGCACTCTTAAGGCCC |
| qRat-Ocm2-R | TGGGTGGTTTTTAAAGGCATTGT |
| qRat-Rcn2-F | ACGAAGAGCAGCAAAGACGA |
| qRat-Rcn2-R | CACGCGGTCATACATCTGGA |
| qRat-Il1rn-F | TGGAAATCTGCAGGGGACC |
| qRat-Il1rn-R | CAGCAATGAGCTGGTTGTTCC |
| qRat-Thop1-F | CAGCCTGTGCTGGGGAC |
| qRat-Thop1-R | AGTGCCTTCAGTGTGCTCTC |
| qRat-Cry61-F | GGGTTTCTAGTGTGGGTCGG |
| qRat-Cry61-R | AACCCGGGCTCCAGTACTAT |
| qRat- Klhl41-F | CCAGATTCCGTCGGTGTTCA |
| qRat- Klhl41-R | GACAGTCGAGAAGAAGCCCC |
| qRat- Pdlim7-F | CCGGGCATTCAGGAGCAG |
| qRat- Pdlim7-R | CACGGATCTTGTTCTGGGCT |
| qRat- Fkbp9-F | TGGCTCCCGGTTTTAATGCT |
| qRat- Fkbp9-R | CACCTTCCCATCTCCGTTCC |
| qRat- Nagk-F | GGAGACGAGGGATCAGCCTA |
| qRat- Nagk-R | ACCTGGAAAGAGGGTCTCCC |
| qRat- Dysf-F | CTGATTTTTCGAGAAGCCAAGATCC |
| qRat- Dysf-R | TGTCTTCCTCTCCTCCTGTGT |
| qRat- Gsdmd-F | AGATCGTGGATCATGCCGTC |
| qRat- Gsdmd-R | GACACTGGTTCTGGAGCACT |
| qRat- C1qtnf5-F | AGCTAGGAGTCTGTCAGCGA |
| qRat- C1qtnf5-R | TAGTCTGTAGGCCCTTCCCG |
| qRat- Rcn3-F | CGGCAGTGAAGTCGGTTACT |
| qRat- Rcn3-R | TCTCAGCTTTGCTCAGTCGG |
| qNDRG1-F | CTCCTGCAAGAGTTTGATGTCC |
| qNDRG1-R | TCATGCCGATGTCATGGTAGG |
| qLANA-F | CCTGGAAGTCCCACAGTGTT |
| qLANA-R | AGACACAGGATGGGATGGAG |
| qK9-F | GTCTCTGCGCCATTCAAAAC |
| qK9-R | CCGGACACGACAACTAAGAA |
| ChIP-GAPDH-F | TACTAGCGGTTTTACGGGCG |
| ChIP-GAPDH-R | TCGAACAGGAGGAGCAGAGAGCGA |
| ChIP-TR-F | GGGGGACCCCGGGCAGCGAG |
| ChIP-TR-R | GGCTCCCCCAAACAGGCTCA |
